# A Review of Gait Analysis Using Gyroscopes and Inertial Measurement Units

**DOI:** 10.3390/s25113481

**Published:** 2025-05-31

**Authors:** Sheng Lin, Kerrie Evans, Dean Hartley, Scott Morrison, Stuart McDonald, Martin Veidt, Gui Wang

**Affiliations:** 1 School of Mechanical and Mining Engineering, The University of Queensland, Brisbane, QLD 4072, Australia; sheng.lin@uq.edu.au (S.L.); kerrie.evans@healthia.com.au (K.E.); dean.hartley@healthia.com.au (D.H.); scott@iorthotics.com.au (S.M.); s.mcdonald1@uq.edu.au (S.M.); m.veidt@uq.edu.au (M.V.); 2 Healthia Limited, Brisbane, QLD 4006, Australia; 3 Faculty of Medicine and Health, The University of Sydney, Sydney, NSW 2050, Australia; 4 iOrthotics Pty Ltd., Brisbane, QLD 4030, Australia; 5 Nantong Institute of Advanced Technology, Shanghai Jiaotong University, Nantong 226300, China; 6 Institute of Rehabilitation Medicine and Health Care, Henan Academy of Innovations in Medical Science, Zhengzhou 450000, China

**Keywords:** gait analysis, gait events, gait phase, inertial measurement units, wearable sensors

## Abstract

Wearable sensors are used in gait analysis to obtain spatiotemporal parameters, with gait events serving as critical markers for foot and lower limb movement. Summarizing detection methods is essential, as accurately identifying gait events and phases are key to deriving precise spatiotemporal parameters through wearable technology. However, a clear understanding of how these sensors, particularly angular velocity and acceleration signals within inertial measurement units, individually or collectively, contribute to the detection of gait events and gait phases is lacking. This review aims to summarize the current state of knowledge on the application for both gyroscopes, with particular emphasis on the role of angular velocity signals, and inertial measurement units with both angular velocity and acceleration signals in identifying gait events, gait phases, and calculating gait spatiotemporal parameters. Gyroscopes remain the primary tool for gait events detection, while inertia measurement units enhance reliability and enable spatiotemporal parameter estimation. Rule-based methods are suitable for controlled environments, whereas machine learning offers flexibility to analyze complex gait conditions. In addition, there is a lack of consensus on optimal sensor configurations for clinical applications. Future research should focus on standardizing sensor configurations and developing robust, adaptable detection methodologies suitable for different gait conditions.

## 1. Introduction

Walking is a complex activity that integrates the musculoskeletal, nervous, cardio-vascular, and respiratory systems to ensure balance, mobility, and stability [1]. Gait serves as an indicator of the health of these interconnected systems, reflecting their functionality and coordination. Impaired gait can result in falls, injuries, and reduced mobility, which can have a significant impact on an individual’s quality of life and independence [2,3]. Consequently, gait analysis is a critical tool which provides insight into general health and specific conditions.

**Figure 1:**
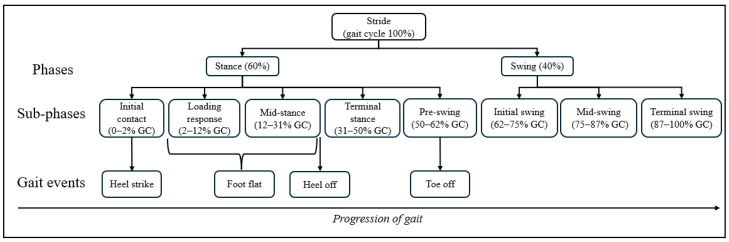
The walking gait cycle and its events [3].

Gait analysis systematically describes individual walking patterns based on the gait cycle [4]. As illustrated in Figure 1, a gait cycle has two main phases: stance phase and swing phase. From a biomechanical perspective, the stance phase can be further subdivided into five sub-phases: initial contact (IC), loading response (LR), mid-stance (MSt), terminal stance (TSt), and pre-swing (PS). The swing phase is subdivided into three sub-phases: initial swing (IS), mid-swing (MSw), and terminal swing (TSw) [5]. Gait events are critical markers in the analysis of walking patterns and describe specific points in relation to movement of the foot during the gait cycle. There are four distinct gait events during the stance phase: heel strike (HS), foot flat (FF), heel off (HO), and toe off (TO). Gait events provide critical spatiotemporal data on foot and lower limb movement during locomotion, serving as key indicator for analyzing lower limb mechanics and producing physical quantities of gait, such as stride length, stride time, and velocity. Definitions and abbreviations for gait events and phases are available in Supplementary Material Table S1.

**Figure 2:**
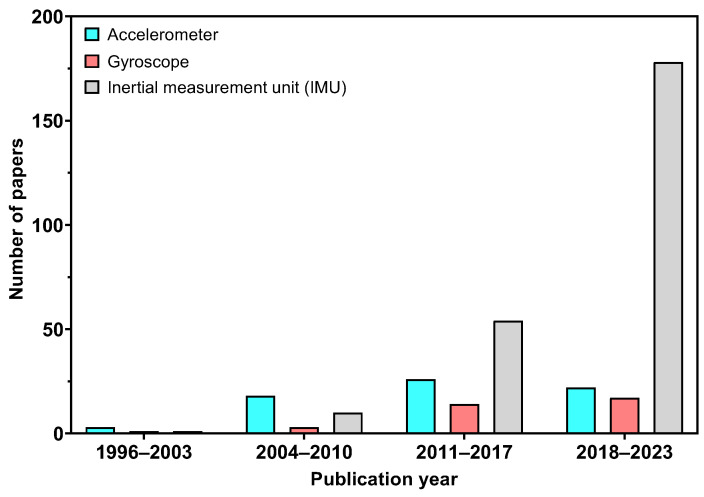
Number of publications in initial investigations using Web of Science.

The utilization of quantitative instruments in gait analysis, which has been accessible for several decades, is predominantly limited to research institutions and is not extensively utilized in clinical environments [6,7,8]. Such instruments, like motion capture systems, are primarily restricted due to their high costs and intricate nature, the complexity of the protocols and data analysis processes inherent in conventional gait laboratories, as well as the varied training, experience, and preferences of clinical personnel [9,10]. With the advantages of sensing and information technologies, wearable sensors have become an integral tool in gait analysis [11]. These sensors, like gyroscopes and inertial measurement units (IMUs), have been used to collect kinematic data in gait analysis. Due to their small size, relatively low cost, and their ability to output data that captures motions in three dimensions, they can replace or complement motion capture systems, and there is a clear trend toward developing wearables suitable for clinical environments [12,13]. Gyroscopes are capable of detecting angular velocity and orientation, showing periodic and repeatable patterns during the gait cycle. IMUs, the most commonly used wearable sensor [14], typically consist of an accelerometer, gyroscope and a magnetometer allowing researchers to compute spatiotemporal parameters. Capturing acceleration and angular velocity allows the measurement of different gait parameters like walking speed, step and stride length, which can be evaluated and compared between different populations. Since the 1990s, considerable research has focused on the use of wearable sensors to investigate gait [15]. Figure 2 illustrates the number of publications vs. year, demonstrating the increasing trend of using wearable sensors in gait research. As shown in Figure 2, accelerometers were predominantly used in gait studies until 2011, but their use has declined over the past 10 years. The use of gyroscopes and IMUs in gait studies became increasingly prevalent between 2011–2017 and IMU sensors have dominated gait research over the last 10 years.

Previous reviews on wearable sensors have focused on various aspects, including gait in running conditions [16,17], pathological gait [18,19,20,21,22], fall risk [23,24], and rehabilitation [19,25]. One review summarizes the technical path for obtaining spatiotemporal parameters using IMUs [26]. Furthermore, there have been a number of reviews focusing on the use of accelerometers [27,28], IMUs [29,30,31], and in one case in combination with pressure sensors [32] in gait analysis. Several reviews have addressed gait events or gait phases using wearable sensors for different purposes [28,29,31,33,34]. For example, to detect gait events or gait phases using multiple sensors and sensor combinations [28,33], and real-time gait acquisition and artificial intelligence [31,34]. By examining the above reviews, it is evident that a clear understanding of how angular velocity and acceleration signals individually or collectively contribute to gait event and gait phase detection is still lacking. Specifically, no existing review has addressed how these two types of signals are combined to precisely identify gait events and phases or explicitly summarize the exact locations of these events within the sensor signals themselves. Furthermore, an analysis highlighting the similarities and differences among various gait events and gait phases detection methods, calculation of gait spatiotemporal parameters methods, remains absent from current literature.

Given the increased use of wearable sensors, particularly gyroscopes and IMUs, in gait analysis, it is critical to clarify how angular velocity signals from gyroscopes and the combination of angular velocity and acceleration signals from IMUs individually or collectively contribute to the identification of gait events, gait phases, and subsequent calculation of spatiotemporal parameters. Although both angular velocity and acceleration signals are integral components of IMUs, literature often lacks explicit differentiation regarding their specific roles in gait analysis. Therefore, the primary objective of this review is to summarize the existing state of knowledge on how angular velocity signals (from gyroscopes) or combined angular velocity and acceleration signals (from IMUs) facilitate gait event detection, gait phase identification, and quantification of gait spatiotemporal parameters. Additionally, the review aims to provide an overview of current practices in sensor specifications and configurations, discuss methodological strengths and limitations, and propose potential directions for future research and clinical applications.

## 2. Review Methodology

### 2.1. Search Strategy and Selection Criteria

A literature search was conducted in July 2024 using the Web of Science, Scopus, and PubMed as the primary databases and using the PRISMA (Preferred Reporting Items for Systematic Reviews and Meta-Analyses) guidelines. Based on our survey, there was a significant surge in the number of studies utilizing gyroscopes and IMUs for gait analysis between 2011 and 2017. Consequently, we limited our search to papers published within the period of 2014–2025 to focus on the most recent advancements while maintaining a 10-year timespan to ensure a comprehensive yet manageable review of the literature. In order to identify the target papers, the following search command were used to retrieve the desired results: (ALL = (IMU) AND (ALL = (gait events) OR ALL = (gait phase))), (ALL = (IMU) AND (ALL = (gait events) OR ALL = (gait detection))), (ALL = (Gyroscope) AND (ALL = (gait events) OR ALL = (gait phase))), ALL = (IMU) AND (ALL = (“heel strike”) OR ALL = (“toe off”) OR ALL = (“heel off”) OR ALL = (“foot flat”)). Following the completion of the searches, all references were imported into the Rayyan platform [35] to remove duplicate papers.

The screening procedure was completed independently by two of the authors of this study. After removing duplicates, the two authors screened articles based on titles and abstracts to identify those meeting the eligibility criteria. If the title and abstract lacked sufficient information, the full text of the article was reviewed. In cases where there was disagreement between the two authors and consensus could not be reached through discussion, a third author was consulted and made the final decision.

### 2.2. Eligibility Criteria

Studies were included if IMUs or gyroscopes were utilized as primary measurement tools and had a clear description of the methods used to identify gait events or gait phases. The identification methods could be presented in various forms, including mathematical formulas, algorithmic flowcharts describing the procedure of gait event or gait phase detection, or illustrative figures marking gait events on sensor-derived signals. Studies with main objective of determining gait events and gait phases from IMU or gyroscope and studies that explicitly reported the use of IMU or gyroscope signals for calculating spatiotemporal gait parameters were also eligible for inclusion.

## 3. Results

Table 1 provides a comprehensive summary of all 66 articles, covering sensor type, location, sampling frequency, filter settings, etc. The result sections are organized as follows: Section 3.1 and Section 3.2 describe the specification of sensors, numbers, and location of sensors in papers; Section 3.3 introduces common methods for sensor synchronization; Section 3.4 provides an overview of gait events detection. Section 3.5 and Section 3.6 provide a detailed description of the methodology applied using rule-based and machine learning. Section 3.7 gives examples of instruments and methods of validation. Section 3.8 and Section 3.9 describe how to obtain gait physical quantities and the application of gait measurements, respectively.

### 3.1. Specification of Sensors Used in Papers

The sampling frequency of the sensors determines how well the gait pattern is reproduced during data acquisition. In total, 52 articles reported the sampling frequency of the sensors. In these studies, the sampling frequency ranged between 25 Hz [36] and 1500 Hz [37]. Of the articles, 42 reported sampling frequencies between 100 Hz and 200 Hz. Of these, the majority of studies used a sampling frequency of 100 Hz (*n* = 30). Some studies used a sampling frequency of less than 100 Hz (*n* = 12), and a small number of studies used a sampling frequency of more than 200 Hz (*n* = 7).

**Figure 3:**
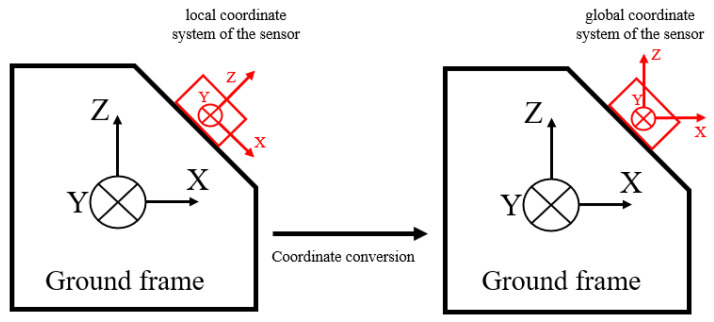
Coordinate conversion of IMU.

Coordinate conversion depends on the type of data output from the IMU sensors. For an IMU capable of providing quaternion or three-axis orientation angles as outputs, the coordinate transformation can be directly performed. However, for an IMU that provides only raw accelerations and angular velocities, direct integration of angular velocities to estimate angles typically leads to signal drift. Consequently, sensor fusion algorithms, such as Kalman or Madgwick filters, become essential for estimating the initial orientation angles [38,39]. As illustrated in Figure 3, coordinate conversion involves transforming acceleration data from the local sensor coordinate frame to a global reference frame. This transformation is usually performed using rotation matrices or quaternion-based methods and is crucial for accurately calculating spatiotemporal gait parameters in subsequent analyses.

Due to the high sensitivity and the generally high level of signal to noise ratio of wearable sensors, filter techniques are used to eliminate disturbances and smooth off sensor signals. A total of 32 articles have reported on the types of filters they used. The filter retains the important low-frequency components of the gait signal by removing high-frequency noise from the environment or vibration with the aim to make the signal smooth and more accurate. The vast majority of signal processing used low-pass filters (*n* = 21) to deal with noise in accelerometer and gyroscope signals. The most frequently mentioned filter was the Butterworth filter (*n* = 21). The settings for the Butterworth filter varied in the articles in terms of order and cut-off frequency. The main reported orders ranged from the first to eighth order, and the cut-off frequency ranged from 0.0002 to 40 Hz. Other types of sensors include the Savitzky–Golay filter [40], moving average filter [41], interpolation filter [42], and Gaussian filter [43].

### 3.2. Location of Sensor Placement and Numbers

Most of the articles included in this review provided details on where the sensors were attached. The sensor locations were mostly on the shank (*n* = 42), followed by the thigh (*n* = 32), and the dorsum of the foot (*n* = 27). Only two studies reported that sensors were attached to the heel of the foot. Sensors placed on the shank and thigh were also categorized into lateral (*n* = 15) and frontal (*n* = 14) orientations.

The number of sensors placed on the lower limb varied among the studies. Most studies placed two sensors (*n* = 31), followed by four sensors (*n* = 11), one sensor (*n* = 8), and three sensors (*n* = 6). A total of 12 studies used more than four sensors, and one study used 12 sensors on the lower extremity [44].

### 3.3. Sensor Synchronization

Most of the studies involved the application of multiple IMU sensors or combinations of IMU and other sensor types for gait event detection or gait analysis. Therefore, data synchronization between sensor systems becomes a critical issue. Based on investigation, several methods of sensor synchronization were identified. The first and most common method employs commercial sensor systems, which typically include dedicated software that supports synchronization of multiple IMUs or integration of IMUs with other sensors [45,46]. This simplifies the synchronization process and reduces the need for manual timestamp alignment. The second method utilizes time synchronization protocols in sensor networks, establishing a unified time reference between IMUs or different systems through Bluetooth, triggers, and WIFI networks [47]. Lastly, an event-based synchronization approach was employed by several studies [48,49,50], providing recognizable signals in both IMU and pressure sensor data, allowing precise timestamp alignment across sensor types.

### 3.4. Overview of Gait Events Detection

It is important to note that different studies adopted different rules or methods to identify gait events. The following content aims to provide general methods used in the detection of gait events and phases.

Rule-based methods are mostly time-domain analyses that involve the application of multiple predefined constraints on signals to extract gait features. Gait is recognized as a stable and quasi-periodic cyclic phenomenon that delineates the pattern of bipedal locomotion [51]. For example, the stance phase is usually defined as stable while the swing phase is considered as quasi-periodic in both gyroscope and acceleration signals. A quasi-periodic waveform is characterized as a signal exhibiting approximate periodicity, with its period subject to variation within a certain range. While quasi-periodic waveforms seem to recur at regular intervals, each cycle does not maintain a uniform length. To facilitate the utilization of rule-based methods in quasi-periodic wavelength signals, one common approach is to set up a threshold for the sensor output signal. The known threshold techniques can be categorized into fixed and adaptive thresholds [40]. Fixed threshold refers to the use of a constant threshold across the entire dataset, whereas adaptive thresholds involve the dynamic adjustment of the threshold based on real-time data to accommodate varying environmental conditions and individual differences.

The second method is the peak heuristic algorithm, which is based on detecting peak points in a signal to correspond to important events or features. Peak points are typically identified as local maxima or minima within a specified window period. A significant proportion of contemporary methodologies rely on heuristic strategies, particularly for the purpose of phase segmentation, e.g., [50].

The third approach is based on mechanical models with one or more joints and linkages [52,53]. The inverted pendulum model is a simple single-jointed mechanical model used to simulate the forward movement of the body with a fixed foot in stance or the foot with a fixed body in swing in the sagittal plane [52]. The dynamic walking model, similar to the inverted pendulum model, also in the sagittal plane, allows for more detailed calculation of relative motion by defining upper body, thigh, lower legs, and feet [53]. In both cases, the physical quantities of gait can be obtained in the time domain of the gait cycle with the constraints of movement.

Machine learning is an alternative methodology for conducting gait analysis to derive gait-related physical quantities. Machine learning is a subset of artificial intelligence (AI) that allows computers and systems to learn from data and improve their performance. It relies on statistical techniques and identifies patterns and relationships within data, enabling the system to make predictions, decisions, or classifications based on new and unseen data. In gait analysis, machine learning techniques are employed to automatically analyze gait data and extract meaningful gait quantities or identify specific events in the gait cycle. A total of 13 types of machine learning models have been reported to determine the occurrence of gait events in the literature. Three categories of models can be classified based on the characteristics of the input data: temporal data models, image or spatial data models, and classification or regression models.

The temporal data model includes the Hidden Markov Model (HMM), Long Short-Term Memory (LSTM), bi-directional Long Short-Term Memory (bi-LSTM) and Temporal Convolutional Network (TCN). Time-series models are based on time-varying data and establish relationships between data points to make predictions. HMM and LSTM were employed in gait studies that utilize gyroscopic signals as input. HMM served as a robust statistical model for the classification of time series, wherein an unobserved process was inferred from observable data produced by stochastic processes [54]. LSTM, a specialized variant of recurrent neural networks, was tailored to efficiently handle and predict long-term dependencies in data sequences [42].

Image or spatial data models include Convolutional Neural Network (CNN), Fully Convolutional Network (FCN), and Deep Convolutional Neural Networks (DCNN). These models classify or predict the spatial structure of data from 3D data by local feature extraction and layer-by-layer construction of features. CNN is a prevalent spatial data model that produces translationally invariant features, ensuring time-invariance in time-series regression. This enables a resilient gait phase estimator adept at managing temporal variations in sensor data due to diverse walking velocities and alterations in key gait event timings throughout the gait cycle [55].

Classification or regression models include Support Vector Machine (SVM), Quadratic Discriminant Analysis (QDA), Random Forest, and Decision Trees. Classification models were used to predict discrete labels. For example, using SVM to classify healthy and diseased gaits [56,57]. Regression models, on the other hand, were used to predict continuous values from a decision tree to identify gait events using different time windows [58].

**Table 1:** Summary data of all reviewed papers.

Ref.	Sensor Types *	Sensor Location	Number	Sample Frequency	Signal Filter	Method Category	Type of Machine Learning	Target Population
[59]	IMU, ZurichMove, Zürich, Switzerland	Wrist, pelvis, foot, head	4	200 Hz	N/A	Machine learning	Convolutional neural networks (CNN) and temporal convolutional networks (TCN)	79 healthy adults
[60]	IMU, Xsens MTw sensors Enschede, The Netherlands	Back waist, lateral thigh and shank, dorsum of the foot	7	100 Hz	4 order Butterworth low-pass filter with 5 Hz cut-off frequency	Rule-based method		20 healthy adults
[61]	IMU, EBIMU-9DOFV5; E2BOX, Seoul, Republic of Korea	Lateral thigh	1	100 Hz	2 order Butterworth band-pass filter main frequency ±0.5 Hz for acceleration, 2 order Butterworth filter with a 3 Hz cutoff frequency for angular velocity	Rule-based method		2 healthy adults; 1 patient with left hemiplegia;
[62]	IMU, Xsens MTw sensors Enschede, The Netherlands	Foot	2	100 Hz	N/A	Rule-based method		12 healthy adults and 12 stroke patients
[63]	IMU, MTw Awinda XSens, The Netherlands	Dorsum of foot	2	100 Hz	8 order low-pass Butterworth filter with a 14 Hz cut-off frequency	Rule-based method		13 healthy adults; 29 patients with multiple sclerosis (MS), and 21 post-stroke hemiplegic patients with spastic equino varus foot (EVF)
[64]	IMU, MyoMotion, NORAXON, USA	sacrum, frontal thigh and shank, foot	4	100 Hz	N/A	Machine learning	1D CNN-Bi-LSTM	26 healthy adults
[36]	IMU	Thigh and shank	2	25 Hz	N/A	Machine learning	Deep Learning (DNN, Recursive Neural Network (RNN), LSTM)	10 healthy adults
[55]	IMU, Xsens, Enschede, The Netherlands	Lateral mid-thigh and shank	4	100 Hz	4 order Butterworth low-pass filter with a 10 Hz cut-off frequency	Machine learning	Convolutional neural network (CNN)	16 healthy adults
[45]	IMU (gyroscope), Vicon Blue Trident, UK	Lateral shank	2	225 Hz	4 order Butterworth low-pass filter with a 10 Hz cut-off frequency	Rule-based method		15 healthy adults
[65]	IMU, Invensense, San Jose, CA, USA	Frontal thighs and shanks	4	500 Hz	N/A	Machine learning	Artificial neural networks (ANN)	N/A (based on Benchmark ENABL3S Dataset)
[66]	IMU, BMI270 BOSCH, Germany	Dorsum of foot	2	73.5 Hz	5 order Butterworth low-pass filter with a 4 Hz cut-off frequency	Machine learning	Support vector machine (SVM)	32 healthy adults
[67]	IMU, Bionic Pro (Motesque, Germany), SageMotion (SageMotion, USA),	Lateral lower legs	2	500 Hz (Bionic Pro), 100 Hz (SageMotion)	Low-pass Butterworth filter with 15 Hz cut-off frequency	Rule-based method		51 healthy adults and 41 stroke patients
[68]	IMU, Xsens, Enschede, The Netherlands	Frontal waist, shank, dorsum of foot	5	100 Hz	N/A	Machine learning	Bi-directional long short-term memory (bi-LSTM)	3 healthy adults
[69]	IMU	Shank, thigh, and waist	6	N/A	N/A	Machine learning	Deep neural network (DNN)	N/A (based on ENABL3S dataset)
[50]	IMU, MetaMotionC, MbientLab, San Fransisco, CA, USA	Shank	2	100 Hz	N/A	Machine learning	Fully convolutional network (FCN)	5 healthy adults
[47]	IMU, MPU 9250, Wit Motion, China	Lateral shank	1	100 Hz	2 order Butterworth filter with 10 Hz cut off frequency	Rule-based method		10 healthy adults
[70]	IMU, SEEED XIAO nRF52840 Sense, China	Lateral shank	2	100 Hz	N/A	Rule-based method		8 healthy adults
[71]	IMU, Adafruit BNO085, USA	Frontal thigh, shank	2	N/A	N/A	Machine learning	Time series forest classifier	N/A
[40]	IMU (gyroscope), INDIP wearable multi-sensor system, Italy	Dorsum of foot	2	100 Hz	Low-pass FIR filter, n = 120 coefficients, 3.2 Hz cut off frequency, Savitzky–Golay filter	Rule-based method		18 healthy adults
[51]	IMU, MPU9250, InvenSense, Japan	Frontal thigh, shank, dorsum of foot	3	100 Hz	Mean filtering algorithm	Machine learning	Hidden Markov model (HMM)	17 healthy adults
[72]	IMU, JY901, China	Lateral thigh, shank, dorsum of foot	6	100 Hz	N/A	Machine learning	Data pre-filtering long short-term memory and convolutional neural network (DPF-LSTM-CNN)	20 healthy adults
[44]	IMU, Delsys Trigno IM, Delsys Inc., Boston, MA, USA	Gluteus maximus, biceps femoris, vastus lateralis, gastrocnemius, tibialis anterior, erector spinae, rectus abdominus, dorsum of foot	16	148 Hz	N/A	Machine learning	Random forest, long short-term memory (LSTM), bi-directional LSTM (bi-LSTM)	30 healthy adults
[42]	Gyroscope, MPU6050, USA	Frontal thigh, shank	2	1000 Hz	interpolation filtering	Machine learning	Long short term memory (LSTM)	7 healthy adults
[38]	IMU	Dorsum of foot	1	200 Hz	Low-pass filter	Rule-based method		N/A
[73]	IMU, MPU9250 InvenSense, Japan	Hip, left/right thigh, left/right shank, and left/right foot	7	200 Hz	N/A	Machine learning	Temporal convolutional network (TCN), Long short term memory (LSTM)	10 healthy adults
[74]	IMU, Xsens MTw Awinda, The Netherlands	Lateral thigh, thigh pocket, frontal shank, dorsum of foot	4	100 Hz	2 order Butterworth filter with a cut-off frequency of 15 Hz in foot and shank, 1.5 Hz in thigh	Machine learning	Hidden Markov model (HMM)	9 healthy adults
[58]	IMU, Physilog 5, GaitUp Ltd., Lausanne, Switzerland	Dorsum of foot	1	512 Hz	N/A	Machine learning	Decision trees	40 healthy adults
[75]	IMU, ZurichMOVE, Switzerland	Lateral ankle	2	200 Hz	1 order high-pass Butterworth filter with a 0.0002 Hz cut-off frequency	Rule-based method		17 healthy adults; 10 patients with spinal cord injury
[76]	IMU, BOSCH BMI family, Germany	Frontal thigh, shank, dorsum of foot	3	N/A	N/A	Machine learning	Long short term memory (LSTM)	2 healthy adults
[77]	IMU, IMU-Z2, ZMP Inc., Tokyo, Japan	Trunk, the frontal thighs, shank	5	100 Hz	5 order low-pass Butterworth filter with 2 Hz cut off frequency	Rule-based method		7 healthy adults; 15 patients with knee osteoarthritis
[43]	IMU, Xsens MTw Awinda, Xsens Technologies B.V., Enschede, The Netherlands	Lateral ankle	2	100 Hz	Gaussian smoothing filter	Machine learning	Long short term memory (LSTM)	16 healthy adults; 11 patients with glaucoma and 37 patients with chronic low back pain
[78]	IMU, Noraxon U.S.A. Inc., Scottsdale, Arizona, USA	Lateral shank	1	200 Hz	High-pass IIR filter with 0.15 Hz cut off frequency, 4 order Butterworth filter with a 10 Hz cut-off frequency	Rule-based method		11 healthy adults; 14 patients with Parkinson and 9 patients with stroke
[79]	IMU (gyroscope), MTw Awinda, Xsens, Enschede, The Netherlands	Lateral thigh	2	100 Hz	2 order Butterworth filter with 10 Hz cut off frequency	Rule-based method		10 healthy adults; 8 patients with chronic phase after stroke
[80]	IMU (gyroscope), Shimmer Research, Dublin, Ireland	Frontal thigh, shank	4	51.2 Hz	low-pass Butterworth filter with a 5 Hz cut-off frequency	Rule-based method		12 healthy adults; 13 patients with Parkinson
[81]	IMU, MEMS PA-GS, China	Dorsum of foot	1	100 Hz	N/A	Rule-based method		10 healthy adults
[82]	IMU, Invensense, Sunnyvale, CA, USA	Dorsum of foot	1	100 Hz	N/A	Machine learning	Hidden Markov model (HMM)	8 healthy adults
[41]	IMU, PABLO Tyromotion GmbH, Graz, Austria	Dorsum of foot	2	110 Hz	Moving average filter	Rule-based method		39 healthy adults
[83]	IMU, Xsens MTw, Enschede, Netherlands, Opal, V2 APDM Inc., Portland, OR, USA)	Trunk, shank	3	60, 75,100 Hz	N/A	Rule-based method		10 healthy adults; 10 patients with vestibular neuritis
[57]	IMU, MPU-6050 InvenSense, USA	Lateral shank	2	100 Hz	N/A	Machine learning	Support vector machine (SVM)	13 healthy adults; 8 patients with peripheral neuropathy, 13 patients with post-stroke, 15 patients with Parkinson
[84]	IMU, XSens^®^ Technologies, Enschede, the Netherlands	Dorsum of foot	2	100 Hz	N/A	Rule-based method		10 healthy adults; 22 patients with progressive multiple sclerosis
[85]	IMU, Myon/Cometa aktos-T, Milan, Italy	Pelvis, thighs, shank, dorsum of foot	7	50 Hz	N/A	Machine learning	Deep convolutional neural networks (DCNN)	12 healthy adults
[86]	IMU, ZurichMOVE, Switzerland	Shank, wrist, chest	5	50 Hz	1 order low pass Butterworth filter with a 5 Hz and 12 Hz cut-off frequency for acceleration and angular velocity	Rule-based method		40 healthy adults
[56]	IMU, MPU-6050 InvenSense, Inc. San Jose, CA, USA	Foot heel	2	50 Hz	N/A	Machine learning	Support vector machine (SVM)	4 healthy adults; 6 patients with idiopathic Parkinson
[87]	IMU (gyroscope), MPU-6050 InvenSense, Inc. San Jose, CA, USA	Lateral side of shoe	2	N/A	Moving average filter	Rule-based method		1 healthy adult; 1 patient with chronic post-stroke, and 1 chronic myelopathic
[88]	IMU (gyroscope), Trigno Research+, Delsys, MA, USA	Foot heel	2	148 Hz	4 order low pass Butterworth filter with a 6 Hz cut-off frequency	Rule-based method		6 healthy adults
[53]	IMU, TSND151 ATR-Promotions, Japan	Upper body, thighs, lower legs, and feet	7	N/A	N/A	Mechanical modelling		1 healthy adult
[37]	Gyroscope, ADXRS652, ±250°/s Yaw Rate Gyro, Analog Devices Inc., Norwood, MA, USA	Right leg	1	1500 Hz	4 order Butterworth lowpass filter with a 15 Hz cut-off frequency	Rule-based method		13 healthy adults
[89]	IMU, ADIS16448 Analog Devices, USA	Upper part of the lateral shanks	2	256 Hz	N/A	Rule-based method		97 healthy adults; 4 patients with idiopathic normal pressure hydrocephalus
[90]	IMU	Dorsum of foot	2	100 Hz	2 order low-pass Butterworth filter with 17 Hz and 15 Hz cut-off frequency for accelerometer and gyroscope signals	Machine learning	Hidden Markov model (HMM)	9 healthy adults; 9 patients with hemiparesis
[91]	IMU	Thigh and shank	2	100 Hz	N/A	Machine learning	Quadratic discriminant analysis (QDA) classifier	5 patients with stroke
[92]	IMU, MPU-6150 InvenSense, Inc. San Jose, CA, USA	Frontal thigh and shank	2	100 Hz	N/A	Rule-based method		10 healthy adults
[93]	IMU	Lateral thigh and shank	2	128 Hz	5 order low pass Butterworth filter with a 5 Hz cut-off frequency	Rule-based method		10 healthy adults; 20 patients with total knee arthroplasty
[46]	IMU	Medial side of the right foot	2	100 Hz	N/A	Rule-based method		16 healthy adults
[94]	IMU (gyroscope), APDM, Inc. Oregon, USA	Lateral thigh, medial shank	4	128 Hz	3 order Butterworth filters with cut off at 1 Hz intervals from 2 Hz to 20 Hz	Rule-based method		8 healthy adults
[95]	IMU (gyroscope), MPU-6050, InvenSense, Inc. San Jose, CA, USA	Dorsum of foot	2	100 Hz	digital 1st order low-pass filter low-pass filter with a cut-off frequency of 40 Hz	Rule-based method		10 healthy adults
[96]	IMU, JY901 Wei TeZhiNeng Company, China	Dorsum of foot	2	100 Hz	low-pass filter with a 40 Hz cut-off frequency	Rule-based method		11 healthy adults
[49]	IMU (gyroscope), Xsens MTw sensors Enschede, The Netherlands	Shank	2	100 Hz	low pass finite impulse response filter, with a stop-band attenuation of 60 dB A zero-phase filter	Rule-based method		16 patients with knee arthroplasty
[52]	IMU (gyroscope)	Thigh and shank	4	N/A	N/A	Mechanical modelling		11 healthy adults
[97]	IMU (gyroscope)	Thigh and shank	4	50 Hz	N/A	Rule-based method		11 healthy adults
[98]	IMU	Thigh and shank, waist	6	100 Hz	Low-pass filter for acceleration and high-pass filter for gyroscope	Mechanical modelling		1 healthy adult; 1 patient with stroke, and 1 patient with Parkinson
[99]	IMU, OpalTM, APDM, USA	Lower trunk, ankle	3	128 Hz	N/A	Rule-based method		10 healthy adults
[39]	IMU, MTx Xsens, The Netherlands	Thigh, shank, dorsum of foot	3	100 Hz	N/A	Rule-based method		4 healthy adults
[54]	IMU (gyroscope), XBus Master, Xsens Technologies, The Netherland	Lateral thigh, shank, frontal dorsum of foot	3	60 Hz	Low-pass Butterworth filter with 15 Hz cut-off frequency	Machine learning	Hidden Markov model (HMM)	10 healthy adults
[48]	IMU (gyroscope), ITG3200, InvenSense, InvenSense; Sunnyvale, CA, USA	Frontal shank	2	50 Hz	N/A	Rule-based method		10 healthy adults; 10 patients with Vestibular Neuritis
[100]	IMU (gyroscope)	Frontal shank, mid-thigh	4	40 Hz	N/A	Rule-based method		7 healthy adults; 6 patients with Parkinson
[101]	IMU (gyroscope), Microstrain, Inc, USA	Thigh and shank	4	200 Hz	N/A	Rule-based method		16 healthy adults

* Note: sensor types list the type of sensor used in the article and the specific model. IMU (gyroscope): IMU sensors are used in the article, but only the signals from the gyroscope are used.

### 3.5. Rule-Based Methods

#### 3.5.1. Rule-Based Methods Based on Gyroscopes

In comparison to acceleration signals, the quasi-periodic angular velocity measurements obtained from gyroscopes can provide more salient characteristics for the segmentation of gait phases [39]. Most literature reviewed employs angular velocity signals in the sagittal plane to identify gait events and phases.

**Figure 4:**
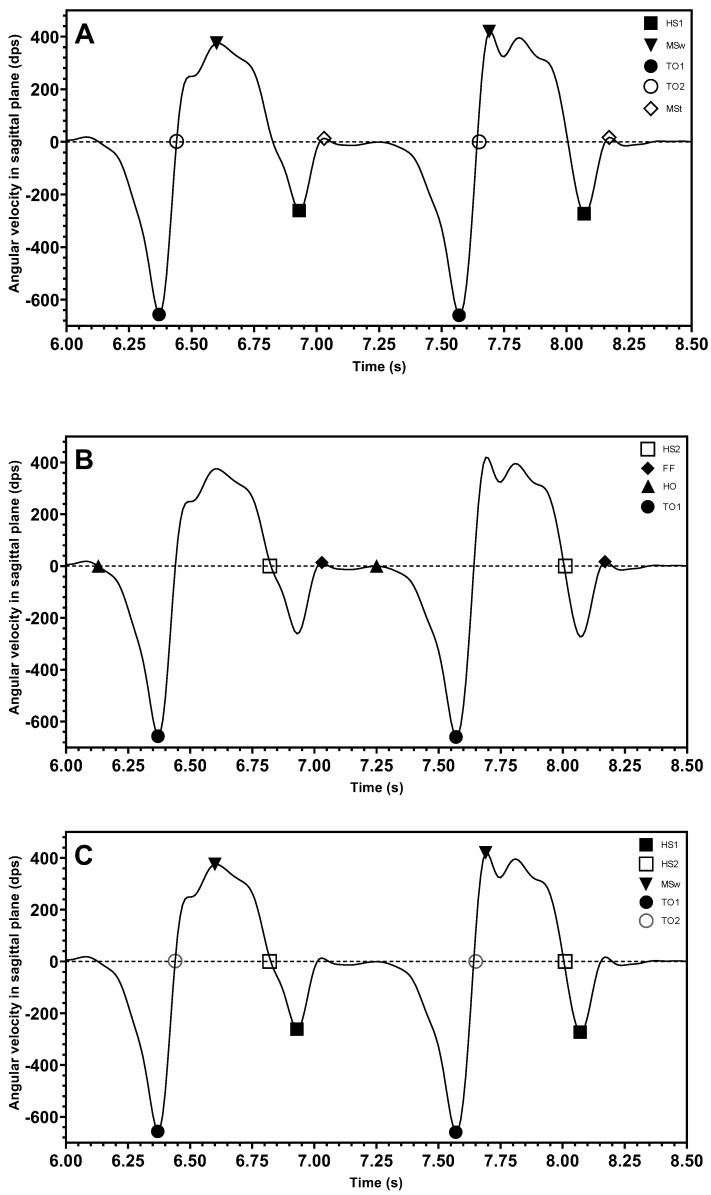
Location of gait events on the shank (**A**), on the dorsum of foot (**B**), and on the foot heel (**C**) (HS1: ■, HS2: □, FF: ♦, HO: ▲, TO1: ●, TO2: ○, MSw: ▼, MSt: ◊).

Most studies consider the peak heuristic algorithm as an important method for determining gait events. Confirmation of the peak values and zero crossings corresponded to the location in the signal where the gait events occur. For example, the three gait events, HS, TO, and MSw, were effectively analyzed using peak heuristic algorithms to determine key points [40,47,48,49,62,70,79,87,94,95,97,101]. However, in different studies, depending on where the sensors were placed, these events were labelled differently on the signal waveform graph. The majority of studies placed sensors on the shank and thigh [37,48,49,94,97,100,101]. One paper reported the sensor placement on the dorsum of the foot [95], and two papers reported having sensors at the heel of the foot [87,88]. Figure 4 shows the example angular velocity signal observed in the sagittal plane in different places, including the sensor attached on the shank in Figure 4A, on the dorsum of the foot in Figure 4B, and on the heel in Figure 4C. The following mathematical formulae summarizes the relationship between gait events and signals in terms of gyroscope output signals from the literature. Here, *ω* represents the angular velocity and *t* is the user-defined detection window time.

When the sensor was placed on the shank, most articles agreed that the frequently detected gait events are the two extreme points corresponding to the maximum value of MSw and the minimum value of TO1 events as expressed in Equations (1) and (2), while HS (expressed in Equation (3)) was considered to be the minimum point of a different window period with a signal amplitude that is larger than the one of the TO1 event.

Toe off 1 (TO1):

(1) $$\omega_{TO1}=\min\left(\omega \left(t\right)\right),\;t\;\epsilon \;\left[t_{{HS}_{last}},\;t_{Mid-swing}\right],\;\omega_{TO1}{<\omega }_{HS1}$$

Mid-swing (MSw):

(2) $$\omega_{MSw}=\max\left(\omega \left(t\right)\right),\;t\;\epsilon \;\left[t_{TO1},\;t_{HS1}\right]$$

Heel strike1 (HS1):

(3) $$\omega_{HS1}=\min\left(\omega \left(t\right)\right),\;t\;\epsilon \;\left[t_{MSw},\;t_{{TO}_{next}}\right]\;$$

However, Allseits et al. [97] cited Bötzel et al. to point out that the TO2 point position should be at the over-zero position after the minimum point and before the maximum point as expressed in Equation (4)

Toe off 2 (TO2):

(4) $$\left\{\;\begin{array}{c} \omega_{TO2}=0,\;{\left.\frac{\mathrm{d}\omega \left(t\right)}{\mathrm{d}t}\right|}_{t=t_{TO2}}>0,\;t_{min}<t_{TO2}<t_{max}\\ t_{min}=argmin\left(\omega \left(t\right)\right)\;which\;t\;\epsilon \;\left[t_{HS1},\;t_{MSw}\right]\\ t_{max}=argmax\left(\omega \left(t\right)\right)\;which\;t\;\epsilon \;\left[t_{{HS}_{current}},\;t_{{HS}_{next}}\right] \end{array}\right.$$

Termination and initiation of forward swing (TOFS, IOFS) were proposed in [100] to identify the swing phase. TOFS occurs at the point 0 where the angular velocity changed from positive to negative, and IOFS occurs at the point 0 where the angular velocity changed from negative to positive. These relations are expressed in Equation (5) and Equation (6), respectively.

Termination of forward swing (TOFS):

(5) $$t_{TOFS}:\;\omega \left(t_{TOFS}\right)=0,\;{\left.\;\frac{\mathrm{d}\omega \left(t\right)}{\mathrm{d}t}\right|}_{t=t_{TOFS}}<0$$

Initiation of forward swing (IOFS):

(6) $$t_{IOFS}:\;\omega \left(t_{IOFS}\right)=0,\;{\left.\;\frac{\mathrm{d}\omega \left(t\right)}{\mathrm{d}t}\right|}_{t=t_{IOFS}}>0$$

The occurrence of MSt was usually at the maximum value between the window times of HS1 and TO1 and was the maximum point in the middle of the negative angular velocity region close to zero [97] as expressed in Equation (7).

Mid-stance (MSt):

(7) $$\omega_{MSt}=\min\left(\omega \left(t\right)\right)≅0,\;t\;\epsilon \;\left[t_{HS1},\;t_{TO1}\right]$$

When the sensor was placed in the dorsum of foot, the positions of the two-gait events, including FF and HO, were also indicated in the waveform graphs. FF was defined as the instant when the signal becomes constant with an amplitude close to zero. HO was defined as the time when the angular velocity becomes negative after FF occurred [95]. The expressions for FF and HO events are defined below in Equations (8) and (9).

Foot flat (FF):

(8) $$\left|\omega \left(t\right)\right|<\epsilon ,\;\left|\frac{\mathrm{d}\omega \left(t\right)}{\mathrm{d}t}\right|\approx 0,\;t\;\epsilon \;\left[t_{HS2},\;t_{TO1}\right]$$

Heel off (HO):

(9) $$\omega \left(t_{HO}\right)<0,\;{\left.\frac{\mathrm{d}\omega \left(t\right)}{\mathrm{d}t}\right|}_{t=t_{HO}}>0,\;t\;\epsilon \;\left[t_{FF},\;t_{TO1}\right]$$

The location of HS2 events was identified to occur after the maximum value of the angular velocity and before the next negative extreme at the point when the signal just passed zero, Equation (10).

Heel strike2 (HS2):

(10) $$\omega \left(t_{HS2}\right)=0,\;{\left.\;\frac{\mathrm{d}\omega \left(t\right)}{\mathrm{d}t}\right|}_{t=t_{HS2}}<0,\;t\;\epsilon \;\left[t_{TO1},\;t_{FF}\right]$$

Hutabarat et al. [88] and Pérez-Ibarra et al. [87] had different interpretations of the position when the sensor was placed in the heel region. Hutabarat et al. considered that the position of the IC with TO occurred at the negative peak value of the angular velocity, which corresponds to the expression in Equations (1) and (3). Meanwhile, Pérez-Ibarra et al. believed that the position of HS with TO occurred at the position past the zero point, which is expressed in Equations (4) and (10). Both agreed that the position of MSw occurred at the extreme value of angular velocity.

Gait phases or events can be effectively determined using angular velocity signals from gyroscopes, specifically by the algorithm proposed by Allseits et al. [97]. In the proposed algorithm, the state of the current gait phase is determined by searching for the occurrence of zero crossing points and determining the change in slope. If the slope changed from negative to positive and meets the minimum swing period, it was recorded as a swing to support event. Similarly, if the slope changed to positive and met the minimum support period, it was recorded as a support to swing period.

The algorithm proposed by Figueiredo et al. [95] also included the derivatives of the signals to detect the rise and fall. Two adaptive maximum and minimum thresholds were set for determining gait events. These thresholds were constantly updated based on initial values MAXthr = 0.7 rad/s, MINthr = −2 rad/s and 60% of the maximum and minimum values of the angular velocities of three valid steps. The MSw event was determined to occur when the maximum angular velocity was detected and exceeded the maximum threshold value. A TO event occurred when the minimum angular velocity was detected and fell below the minimum threshold. HO was defined as the point where the angular velocity turned from zero to negative after the FF stabilization period [95].

Fadillioglu first defined MSw by identifying the maximum angular velocity after detecting the zero-crossings for IC and TO. A time-period constraint was added during this process to ensure accurate event identification [37]. Pérez-Ibarra [87], on the other hand, proposed identifying gait events using sagittal angular velocity in combination with resultant angular velocity, setting a threshold on the resultant angular velocity to determine the region of the MSt phase.

#### 3.5.2. Rule-Based Methods Based on IMUs

The combination of accelerometer and gyroscope outputs in an IMU creates various methods for detecting gait events and gait phases. These signals typically use the peak, inflection, and trend signals of both signals to detect HS, FF, and HO events and TO [39,41,46,63,92,96]. Depending on how terminology is used, articles also deal with IC and Final Contact (FC) or Foot contact and foot off (FO) [61,75,78,84,99], and a small number of articles mention Opposite initial contact (OIC) and Opposite final contact (OFO) [61].

**Figure 5:**
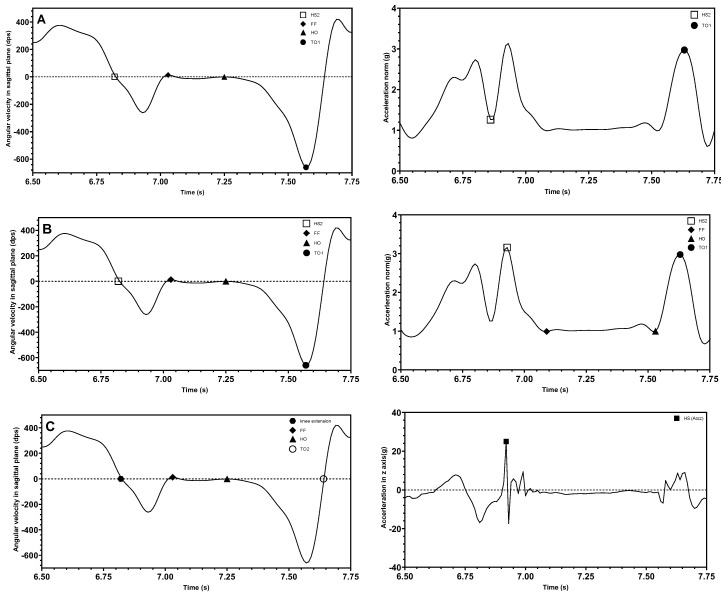
(**A**): Location of gait events on the dorsum of foot gyroscope signal on the sagittal plane (**left**) with norm of acceleration in sagittal plane (**right**). (**B**): On the dorsum of foot gyroscope signal on the sagittal plane (**left**) with norm of acceleration (**right**). (**C**): On the dorsum of foot gyroscope signal on the sagittal plane (**left**) with vertical acceleration (**right**) (HS2: □, FF: ♦, HO: ▲, TO1: ●, HS (Accz): ■).

The combination of IMU signals placed on the dorsum of the foot facilitates the detection of gait events and gait phases [63,96]. Different combinations of waveforms and data characterize gait events in different ways. Several waveform graph combinations described in the literature can be categorized as (1) angular velocity on sagittal plane with norm of acceleration in sagittal plane; (2) angular velocity on sagittal plane with norm of acceleration; or (3) angular velocity on sagittal plane with vertical acceleration signal. Figure 5 illustrates the combination of signal waveforms for each of the three classifications.

The introduction of acceleration signals aids in the identification of some gait events. Different forms of acceleration signals have different formal expressions for recognizing gait events. Shuo et al. [96] used the *x*-axis and *z*-axis of acceleration to calculate the acceleration norm in the sagittal plane, as expressed in Equation (11) and the corresponding signal in Figure 5A, and applied it to determine two gait events, HS and TO. The acceleration norm defined in Equation (12) and shown in Figure 5B is calculated based on the value of the accelerations in all three coordinate directions and was used to determine the four gait events: HS, FF, HO, and TO [63]. The signals calculated from both acceleration norms were filtered using a low-pass filter and were nearly identical in numerical magnitude and trend. Uniaxial acceleration can also be used in conjunction with angular velocity to obtain gait events. Lu et al. [51] used vertical acceleration to measure HS events. The results are shown in Figure 5C.

(11) $$a_{norm-sagittal}=\sqrt{a_{x}^{2}+a_{z}^{2}}$$

(12) $$a_{norm}=\sqrt{a_{x}^{2}+{a_{y}^{2}+a}_{z}^{2}}$$

The following mathematical expressions summarize how the above three cases from the literature determine gait events. The HS event is the easiest gait event to identify using different forms of acceleration data. However, the events result in slightly different locations on the measured signal waveforms. Equation (13) expresses the position of the HS event on the acceleration norm signal in the sagittal plane according to the method used by Shuo et al. [96] who pointed out that the HS can be positioned using the nearby peak signal as the window position.

Therefore, the process first involves finding the time point at which the acceleration norm reaches its maximum and then identifying the minimum acceleration norm before this time point. Before confirming this minimum point as an HS event, it is also necessary to ensure that the corresponding HS event has already been detected in the angular velocity signal, confirming that the acceleration trough indeed corresponds to the HS event.

Heel strike2 ($a_{{HS2}_{norm-sagittal}}$):

(13) $$\left\{\;\begin{array}{c} t_{acc\_max}=argmax\left(\left\|a_{norm-sagittal}(t)\right\|\right)\\ a_{{HS2}_{norm-sagittal}}=argmin\left(\left\|a_{norm-sagittal}\left(t\right)\right\|\right)for\;t_{HS2_{acc}}<t_{acc\_max}\;and\;\left\|a_{norm-sagittal}\left(t\right)\right\|>0\\ t_{HS2\_acc}=t_{HS2} \end{array}\right.$$

In contrast to the position of the HS point as judged by Shuo et al., Voisard et al. [63] considered the HS to be at a position where the extreme value of the acceleration norm occurs as expressed in Equation (14).

Heel strike2 ($a_{{HS2}_{norm}}$):

(14) $$a_{{HS2}_{norm}}=argmax\left(\left\|a_{norm}(t)\right\|\right)$$

Applying the same principle outlined above, the acceleration maximum signal in the vertical direction corresponds to the HS event, as expressed in Equation (15).

Heel strike2 ($a_{{HS}_{vertical}}$):

(15) $$a_{{HS}_{vertical}}=argmax\left(\left\|a_{vertical}(t)\right\|\right)$$

Both Shuo et al. and Voisard et al. determined that the TO event was at the maximum within a certain time window, as expressed in Equation (16), which occurred after the maximum value of acceleration within the gait cycle and was characterized by the amplitude of the peak acceleration being less than the maximum acceleration amplitude.

Toe off (a_toe_off_):

(16) $$\left\{\begin{array}{c} {\;t}_{toe-off}=argmax\left(\left\|a_{norm}(t)\right\|\right),\;for\;t_{toe-off}>t_{HS2(a_{norm})}\\ \left\|a(t_{toeoff})\right\|<a_{a_{norm}}\; \end{array}\right.$$

The acceleration signals of both FF and HO events have similar patterns. During the stance phase, the foot is nearly stationary [51]. Hence, the angular velocity and acceleration signals remain stable. For example, study [41] used signals from both resultant acceleration and resultant gyroscope to determine the location of the FF occurrence. It must be noted that automatic judgement of FF and HO events by any analysis program requires the inclusion of angular velocity data.

Foot flat (FF):

(17) $$\left\{\begin{array}{c} \left\|\omega_{{FF}_{norm}}\left(t\right)\right\|\approx 0,\;for\;t\in \left[t_{HS2},t_{TO1}\right]\\ \;\left\|a_{{FF}_{norm}}\left(t\right)\right\|\approx 1,\;and\;\left\|\frac{d\left\|a_{{FF}_{norm}}\left(t\right)\right\|}{dt}\right\|\approx 0,\;for\;t\in \left[t_{HS2},t_{TO1}\right] \end{array}\right.$$

Heel off (HO):

(18) $$\left\|a_{{HO}_{norm}}\left(t\right)\right\|\approx 1,and\left\|\frac{\left\|a_{{HO}_{norm}}\left(t\right)\right\|}{dt}\right\|\leq 1,fort\in \left[t_{FF},t_{TO1}\right]$$

In addition to detecting IC and FC, Yang et al. used acceleration data from the *x*-axis and *z*-axis, along with sagittal angular velocity on the thigh, to identify four gait events: IC, FC, OIC, and OFC [61]. The TO event was determined when the angular velocity signals fell below a specific threshold, while the IC event was identified at the point of maximum values above the *x*-axis and *z*-axis acceleration thresholds. After IC identification, OTO occurs at the peak acceleration during the window period, while OIC occurs at the point where the filtered angular velocity signal is crossing zero from positive to negative.

Werner et al. [75] also detected IC and TO events using IMU signals from the ankle. They first calculated the angular velocity spectrum in the sagittal plane and obtained the gait cycle using Fourier transform. IC was determined by detecting the acceleration maxima in the anterior and posterior directions within an interval of 5% to 45% of the gait cycle after the MSw, while FC was identified at the midpoint between the local minima of the angular velocity and acceleration in the anterior and posterior directions, within the interval of −35% to −5% of the gait cycle before the MSw.

Storm et al. [99] first identified the MSw location using the shank’s IMU signal as the search window for IC and FC. The IC was considered to be the minimum of the angular velocity in the sagittal plane between the start of the search window and the maximum of the acceleration in the anterior-posterior directions, while FC was defined as the minimum of the forward and backward acceleration within the search window.

### 3.6. Machine Learning Methods

A total of 21 articles used machine learning to obtain gait events. Among the number of machine learning models employed, both LSTM and HMM models were used more frequently than other types of models (*n* = 5). This was followed by SVM (*n* = 3) and bi-LSTM models (*n* = 2). The use of multiple IMUs or IMUs combined with other sensors to predict gait events and gait phases by means of machine learning accounted for the majority of the articles in our results (*n* = 19), while the use of gyroscope as an input (*n* = 2) was in the minority.

#### 3.6.1. General Approach for Machine Learning in Gait Events Detection

**Figure 6:**
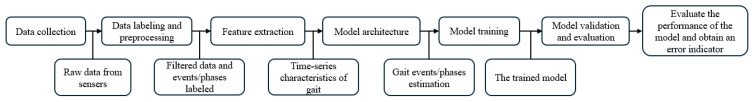
Block diagram flow of gait events/phases prediction using machine learning.

Figure 6 summarizes the general approach for gait events or gait phases acquisition using machine learning. From data collection to feature extraction, the process usually involves one or more sensors. When a single type of sensor was used, one or more of the same sensors were attached to different locations on the lower limbs for data collection. The data from feet mounted sensors were usually used to mark the occurrence of gait events, while data from sensors at other locations were used in the machine learning model for training [43,55,74,76].

When different sensors were used, the combination of multiple devices facilitated better labelling and prediction of gait events. For example, force resistors or force plates placed on the plantar of the feet were used to differentiate between different gait events, while the data from the IMU were fed into machine learning models for training [50,51,54,56,58,66,68,71,72,85,90,91]. Alternatively, a motion capture system was used as the gold standard for labelling gait events [73], and electromyography (EMG) combined with IMU were used as learning inputs to predict angle changes [44].

The format of gait event labelling varied. For example, when gait phases were delineated, gait phases were converted into a binary form to represent HS and TO or stance phase and swing phase [55,66]. However, when multiple gait events were marked, the labelling typically involved identifying temporal critical points [51] or defining multiple class labels [42,43,44,50,54,56,58,68,71,72,73,76,90,91].

The model architecture was customized based on the chosen model. Most of the articles used architectures derived from the original models [43,50,55,56,57,58,66,71,73,74,76,82,90,91] while four articles used variants of these models [44,54,68,85]. A few articles combined models, such as [51], which combined a Gaussian mixture model (GMM) with an HMM model and [72], which combined an LSTM model with a CNN model.

During the data training process, the dataset was usually divided into a training set and a validation set, with the training typically accounting for the majority. Some articles explicitly reported the proportion of the dataset allocated to training versus validation, with slight variations. For instance, 60% of the dataset was used for training and 40% for validation in [68], compared to 70% for training and 30% for validation in [50,58,72,85].

Data validation approaches were mentioned in several articles and can be categorized into two types: cross-validation and direct validation. Most articles which reported cross validation methods primarily used Leave-One-Out Cross Validation (LOOCV). The principle of LOOCV [42,50,54,55,56,57,74,85,90] involves selecting one data as the test set while using remaining data as the training set in each cycle, repeating the process until all data has been tested once. For example, in one study [55], a dataset with 16 sets of participants was used, 14 sets of data were assigned to the training set, one to the validation set, and one to the test set. A different participant was used as the test set in each rotation, looping 16 times. Another common method was 10-fold cross validation [91], which was used to train and test the QDA classifier. The dataset was divided into 10 equal parts, with each iteration using one part as the test set and the remaining 9 parts as training set, looping 10 times. The final output was the average of the 10 results, which was used as the performance evaluation of the model.

Direct validation approaches, such as Hold-out validation, were also employed in some articles [58]. In this approach, the entire dataset was randomly divided into training and testing sets, and the model’s performance was evaluated based on this single deviation rather than cyclic cross-validation.

#### 3.6.2. Machine Learning Methods Using Gyroscope and IMU

Machine learning techniques, such as the LSTM-based Transferable Multi-Modal Fusion method (TMMF) [42] and HMM [54], have been effectively used to identify gait events using gyroscope data. The TMMF method integrates data from various sensors, including EMG, gyroscopes, and VR signals, through a multi-modal fusion block and a feature extractor, followed by classification. The preprocessing steps involve filtering and repairing the data, with specific gait phases manually annotated from VR data based on ankle joint motion. The EMG and gyroscope sensors were then used to train the model and predict joint angles, which were validated against VR data as the gold standard [42].

In contrast, the HMM approach subdivides the gait cycle into four phases (FF, HO, HS, and swing phase), using foot switch data to recognize each phase. Low pass filtering of gyroscope signals and time normalization allow for the precise calculation of angular velocity features. The HMM classifier, trained and validated through cross-validation, exhibits high sensitivity and specificity in detecting gait events within a tolerance window. The performance metrics such as True Positive Rate (TPR) and True Negative Rate (TNR) further validate the model’s accuracy in real-time applications.

Machine learning using IMU data has been more dominant in predicting gait events compared to gyroscope-based approaches. For example, Tang [55] built a multi-rate CNN model and used IMU data from different parts of the lower limbs to extract and predict gait phases. IMU data from the foot was used to annotate the occurrence of gait phases, while IMU data from the thighs and calves were used as inputs into the CNN model for training. The model was trained using the optimiser to minimize the mean square error of the gait phase and to avoid overfitting through an early stopping strategy. To verify the generalization ability of the model, one-subject-out cross-validation was used, ensuring the model could accurately adapt to different gait patterns of individuals. The model was evaluated by spatial root-mean-square error and temporal absolute error to ensure high accuracy in gait phase estimation and gait event detection.

Lu [51] used force-sensitive resistors combined with data from multiple IMU sensors on the lower limb to predict gait events using an HMM model. The force-sensitive resistors were used to classify gait phases and label gait events based on the distribution of force-sensitive resistors across the plantar of the foot. Foot IMU data and thigh IMU data were simultaneously used to identify HS events, with knee flexion events also involved in the labelling process. The final values were predicted using two HMM based algorithms: The real time detection algorithm and the model training algorithm, respectively. The real-time detection algorithm processes IMU data every 10 ms, while the training algorithm processed 10 s of data. The training algorithm used historical data to generate gait detection models, which were periodically updated to improve real-time detection accuracy. Model performance was evaluated by calculating precision, recall, and F1-score, indicating accuracy.

### 3.7. Validation Approach

Most studies included information about the equipment and/or methods used to validate gait events (Table 2). Table 2 also summarizes the results in terms of disparity between the experiments and validation methods. Some studies that labelled N/A used other gait metrics or gait spatiotemporal parameters for validation which are outside of the scope of this review.

Studies employing rule-based methodologies to identify gait events and phases typically utilize pressure detection devices (e.g., force plates, force sensitive resistors, pressure mats) and spatiotemporal parameter devices (e.g., motion capture systems) for validation purposes. A select number of studies employed gait video validation [38].

Despite variation in the equipment used across the studies, the most frequently calculated is the temporal error between the validation device and the method used to illustrate accuracy. The temporal errors were mostly in the order of milliseconds, demonstrating good temporal accuracy for methods. For example, Voisard et al. [63] reported median absolute error of 8 ms and 7 ms for TO and HS respectively. Salminen et al. [45] indicated the mean errors for IC and TO events detection were −1.6 ± 6.3 ms and 0.4 ± 9.0 ms respectively.

In the field of machine learning-based gait event detection, the employment of performance metrics has become common practice for validation and performance quantification. These metrics can be divided into two main categories. The first includes metrics for assessing the performance of classification models. Most research associated with machine learning reported accuracy in the paper. For example, Su et al. [85] showed that the accuracy of swing phase detection was 99.3% when using a DCNN model. In addition, precision, recall, F1-score, and macro-F1 scores were also frequently used. Precision and recall measure the ability of the model to predict and classify accurately, respectively. Values close to 1 indicate fewer false positives and misses. The F1-score provides a more comprehensive evaluation when the values of precision and recall conflict. Studies have reported that their proposed models are capable of classifying gait phases; for example, study [73] reported that the precision, recall, and F1-score for classifying gait phases using the TCN-LSTM model were 98.9%, 98.8%, and 98.9%, respectively. The second category includes statistical indicators for assessing model prediction error. The reported methods include spatial root mean square (sRMSE), spatial mean average error, and temporal mean average square [55], mean squared error [50,74], root mean squared error [42,44,58] and mean absolute error [44]. These performance metrics play a crucial role in evaluating the accuracy and reliability of machine learning models for gait event detection, ensuring both classification effectiveness and precise error quantification.

### 3.8. Gait Physical Quantities

Most gait physical quantities are obtained from gait events, except for angle changes. These physical quantities can be classified into three types: temporal [49,88,95,97], spatial [39,41,52,93,98] and functional [88,91,101].

The temporal gait physical quantities include gait cycle time [49,88,95,97], single and double support time [97], and stance and swing time [88]. The following mathematical formulas summarize the methods used in these articles to calculate the temporal parameters of gait phases. Gait cycle time refers to the time between two identical gait events on the ipsilateral foot, which can also be expressed as stride time according to Equation (19). In the literature, gait cycle time has been calculated by measuring the time of HS events on the same foot [95,97], as well as by IC events [49,88].

Gait cycle time (GCT):

(19) $$GCT=t_{{gait\;evnets}_{next}}-t_{{gait\;events}_{current}}$$

Stance and swing time were calculated from the IC and TO times [49,88]. Stance time is defined as the duration between IC and TO within the same gait cycle, while swing time is the interval between TO in the current gait cycle and IC in the flowing cycle. The sum of the stance and swing times equals the stride time, which is also equivalent to the gait cycle time (GCT). Equations (20) and (21) express the time acquisition for the stance and swing phases.

Stance time (T_stance_):

(20) $$T_{stance}=t_{{TO}_{current}}-t_{{IC}_{current}}$$

Swing time (T_swing_):

(21) $$T_{swing}=t_{{IC}_{next}}-t_{{TO}_{current}}$$

The stance phase can also be further subdivided into single leg support and double leg support [88,97]. Double leg support was calculated as the difference between the time of TO or IC of the contralateral leg and the time of IC or TO of the ipsilateral leg. Single leg support was calculated by subtracting twice the double leg support time from the total stance time. Equations (22) and (23) express the mathematical formulas for calculating single and double support time.

Double leg support (T_DLS_):

(22) $$T_{DLS}=t_{{TO}_{R/L}}-t_{{IC}_{L/R}}$$

Single leg support (T_SLS_):

(23) $$T_{SLS}=T_{stance}-2T_{DLS}$$

Spatial gait physical quantities are mainly related to angles, speeds, and distances. Orientation estimation is an essential process for deriving accurate angle information from IMU data. However, direct integration of angular velocity measurements from gyroscopes commonly results in drift errors, which significantly impact measurement accuracy [39]. Sensor fusion techniques have emerged as effective strategies for mitigating these drift errors. Among sensor fusion algorithms discussed in the literature, the Madgwick filter and Kalman filter are the most commonly employed approaches [38,71]. Specifically, the Madgwick filter, categorized as a complementary filter, integrates accelerometer, gyroscope, and magnetometer data using gradient descent algorithms to estimate sensor orientation. Additionally, Kalman filter-based methods, such as the Extended Kalman Filter (EKF) and Error State Kalman Filter (ESKF), have been adopted in studies to achieve real-time orientation estimation while simultaneously correcting integration drift errors [73,77].

Calculating joint rotation angles typically requires placing sensors on both the upper and lower parts of the joint. Zhang [98] calculated the joint rotation using quaternions, as shown in Equation (24), where *q_i_* is the quaternion expression, *t*_4_ represents the real part of the quaternion, and *T* represents the 3D vector of the imaginary part. To calculate the relative rotation of a joint, quaternion data from both sensors are required. For example, the second equation of Equation (24) represents the rotation of the lower sensor 2 relative to the upper sensor 1. The relative rotation angle is obtained by converting the quaternion data into Euler angles.

(24) $$\left\{\begin{array}{c} {\;q}_{i}=\left(t_{4},T\right)=(t_{4},t_{1},t_{2}t_{3})\\ q_{2\to 1}=q_{2}^{-1}\times q_{1} \end{array}\right.$$

Alternatively, the joint angle can be obtained by utilizing the acceleration data from two IMU sensors and calculating the rotation matrix shown in Equation (25) [93]. An initial rotation matrix *R_initial_* is calculated from the triaxial acceleration of femur and tibia before the movement. This rotation matrix is used to transform the value from the coordinate system in the tibia to the coordinate system in the femur, where *A_Tibia_* represents the acceleration vector *A_Tibia_* = [*a_x_*, *a_y_*, *a_z_*]*^T^*. The angle between the two vectors can be calculated from the data once the experiment starts.

(25) $$\left\{\begin{array}{c} A_{Tibia\;in\;Femur}=R_{initial}\times A_{Tibia}\\ \;\theta =\cos^{-1}\left(\frac{{A_{Femur}\times A}_{Tibia\;in\;Femur}}{\left|A_{Femur}\right|\left|A_{Tibia\;in\;Femur}\right|}\right) \end{array}\right.$$

Velocity and distance or position can typically be obtained from accelerometers, which integrated once to obtain velocity and twice to obtain position [38,39,41,62,86]. Equation (26) describes theoretical velocity and position formulas. Here, *V_r_* represents the acquired theoretical velocity, *P_r_* represents the acquired theoretical positions, and *a*_0_ represents the acceleration information. The form of *a*_0_ can be the norm in the global coordinate system according to Equation (12), which usually eliminates the effect of gravity or matrix containing information about three axes. Both *V_r_* and *P_r_* require the initial velocity *V_i_*(0) and the initial positions *P_i_*(0) as input parameters, respectively. But normally, their values were set to 0 [39].

(26) $$\left\{\begin{array}{c} {\;V}_{r}\left(t\right)=V_{i}(0)+\int_{0}^{t}a_{0}\left(t\right)dt\\ {\;P}_{r}\left(t\right)=P_{i}(0)+\int_{0}^{t}V_{r}\left(t\right)dt \end{array}\right.$$

In fact, the direct integration of acceleration leads to significant data drift, resulting in inaccurate velocity measurement. Thus, zero velocity update (ZUPT) methods are applied to acceleration data to eliminate integration drift by reducing the computed *V_r_*(*t*) to zero during the stance phase of gait. Qiu [39] used two mechanisms to determine the moment for ZUPT: the foot was considered to be in the stationary stance phase when the moving variance of the acceleration and the angular velocity energy both fell below specific thresholds. Similarly, Laidig [41], Yamagishi [38], and Wang [62] used the same principle to calculate foot velocity by integrating acceleration and setting the velocity at FF phase to zero. To correct drift error, linear drift correction was applied by linearly interpolating the velocity, ensuring it was set to zero during the FF phase. The position was then obtained by integrating the corrected velocities. In addition to the use of ZUPT combined with acceleration information, Revi [79] used the angles and angular velocities recorded by individual thigh IMUs, extracted the polar paths in phase diagrams and converted the polar paths obtained at each step into the corresponding walking velocities by means of a linear model that had been fitted by a prior regression. This approach avoids the complicated double integration and excessive zero velocity correction steps.

Gait spatiotemporal parameters, such as step length and stride length, can be computed from the positional information obtained from the velocity. Integral studies usually use the positional information to compute the Euclidean parameter to obtain stride length information [41,62]. Equation (27) describes how step length and stride length are calculated using position information. In the calculation of step length, the *P_right_* and *P_left_* represent the position matrix information of the right and left foot, respectively. Based on the definition of step, step length can be interpreted as the horizontal distance between the left and right feet at a certain gait event moment. On the other hand, in the calculation of stride length, *P_foot_* emphasizes the information of a single foot. The formula can be interpreted as the horizontal distance between a certain foot at the same gait event moments before and after. It is important to note that, in both step length and stride length, the matrix represented by P contains information about the X*Y*-axis at the before and after moments, while *z*-axis data is not a contributing factor in the calculation of length.

(27) $$\left\{\begin{array}{c} step\;length=\left\|P_{right}\left(t_{n+1}\right)-P_{left}\left(t_{n}\right)\;\right\|\\ stride\;length=\left\|P_{foot\;}\left(t_{n+1}\right)-P_{foot}\left(t_{n}\right)\;\right\| \end{array}\right.$$

In addition to using position information to calculate gait spatiotemporal parameters, both studies [52,98] used a mechanical model to calculate the speed and stride length. Study [52] employed the Inverted Pendulum Model to calculate step length, stride length, and gait speed. The method determined the average forward speed using the mid-stance angular velocity measured at the shank and thigh. The step length of a single leg was then calculated using this average speed and time *t_Stp_*. Stride length was obtained by summing the step length of the left and right legs. The gait cycle was calculated based on stride length, and the time between two occurrences of the same gait event on the same leg. In Equation (28) [52], *h_s_* represents the sacral height, *t_Stp_* is the time period from the landing of one foot to the landing of the contralateral heel, and *t_Str_* is the time from the landing of one heel to the next landing of the same heel.

(28) $$\left\{\;\begin{array}{c} \bar{V_{com}}=\bar{\omega_{com}}\times h_{s}=(\frac{\omega_{Shank}^{Mst}+\omega_{Thigh}^{Mst}}{2})\times h_{s}\\ StpL=\bar{V_{com}}\times t_{Stp}\\ StrL={StpL}_{left}+{StpL}_{Right}\\ Gait\;cycle\left(GS\right)=\frac{StrL}{t_{str}} \end{array}\right.$$

Further gait metrics exist, like gait symmetry, which is considered an indicator of the degree of gait control as it relates to temporal gait variables between the lower limbs [101]. Gait symmetry is usually expressed as ratio of the difference between two independent gait qualities. Equation (29) shows the expression of the gait symmetry index, where *X*_1_ and *X*_2_ represent the same gait physical quantity for different samples. For example, Gouwanda calculated the symmetry index (SI) using gyroscope data by comparing the angular velocities of left and right feet during three gait events: HS, MSw, and TO [101]. Similarly, both Hutabarat [88] and Lou [91] calculated a symmetry index based on the timing of the stance phase.

Gait symmetry index (SI)

(29) $$SI=\frac{\left(X_{1}-X_{2}\right)}{0.5(X_{1}+X_{2})}*100\%$$

In summary, various methods have been proposed in the literature to calculate temporal, spatial, and functional gait parameters using IMU and gyroscope sensor data. Despite differences in algorithms and mathematical models, these methods consistently highlight the value and versatility of wearable sensor data in deriving critical gait metrics.

### 3.9. Application of Gait Measurement

Gait analysis has been widely used in disease detection. Differences in gait physical quantities between individuals with pathological gait and healthy individuals have been used to characterize disease progression. A total of 21 articles reported pathological gait. The most frequently mentioned conditions were Parkinson’s (*n* = 7), hemiplegia (*n* = 6), and knee arthritis (*n* = 3).

Neurological disorders tend to alter a patient’s gait pattern. Several articles [56,57,78,80,83,98,100] refer to the use of sensors to detect physical quantities of gait in Parkinson’s disease. Gait characteristics of Parkinson’s patients were measured by the time taken for gait events to occur [56,57,78,83,100] or by stride length [57,83,100], and were compared with those of healthy individuals. For example, one study [100] measured stride time, stride time variability, and stride length by computing angular velocity in the calf and thigh to differentiate gait characteristics between Parkinson’s patients and healthy individuals. Quantitative tools, such as continuous relative phasing [80], analyzed lower limb coordination by calculating phase angle data from angular velocities, further distinguishing gait differences between healthy people and Parkinson’s patients.

Other neurological disorders, such as multiple sclerosis [84] and spinal cord injury [75,87], were also studied to observe gait variability due to disease, using measures like stride length and stride duration. For example, patients with spinal cord injuries exhibited increased step width, decreased step height, and shortened stride length in their gait [75].

Hemiplegia symptoms, often caused by conditions like stroke, affect a patient’s gait. IMU motion data from the thigh or instep have been used to detect stance, swing phases, and the duration of gait events [61,63,79,87,90,91], as well as gait symmetry [61,79], to distinguish gait characteristics between healthy individuals and those with hemiplegia. Revi [79] extracted gait information such as step length, gait cycle time, stance, swing time, and gait symmetry using phase portrait and found that patients with hemiplegia typically exhibit features like asymmetrical gait length, shortened swing period, and prolonged support period. Healthy individuals generally have higher walking speed and longer stride length, whereas post-stroke hemiplegic patients exhibit reduced walking speeds and shortened stride length due to muscle weakness or diminished control [79].

Gait variability in the context of joint injuries is typically measured through changes in angle or angular velocity. Knee osteoarthritis, a common joint disorder of the lower extremities, leads to decreased mobility as the disease progresses. Patients with valgus knee osteoarthritis were tested for lateral acceleration and valgus angular velocity using IMU sensors placed on the thigh and calf. Lateral acceleration detected the lateral motion caused by the knee’s valgus during the stance phase, while valgus angular velocity captured the angular changes during valgus. The results showed that lateral acceleration and valgus angular velocity data were higher in patients with severe osteoarthritis than in healthy individuals [77].

Total knee arthroplasty is one of the treatments for severe knee osteoarthritis. Studies have analyzed post-surgery knee recovery by measuring flexion angle or angular velocity during stance and swing phase [49,93].

## 4. Discussion

### 4.1. Advantages and Challenges in Using Gyroscope and IMU

Combing both IMU and gyroscope data improves the ability to measure the parameter of gait. IMUs are sensitive to gravity, requiring precise calibration, coordinate conversion, and de-gravitation before they can be used in gait event detection. In contrast, gyroscopes are less affected by gravity, making them ideal for detecting angular velocity change, which is critical for identifying events such as HS, TO, and MSw at various locations on the lower limbs. However, gyroscope data may lack sufficient detail for capturing certain events, such as FF and HO. Accelerometers in IMUs, being highly sensitive to linear acceleration change, are more effective at detecting events like HS. Certain events such as FF can also be detected by combining gyroscope signal features when the foot remains stationary for a short period. The IMU generates six axes of signal data, from which acceleration and angular velocity norms can be calculated. Although most research focuses on using the *z*-axis of acceleration signal or angular velocity in the sagittal plane to determine gait events, the versatility of IMU data allows for various combinations to enhance the accuracy and robustness of gait event detection.

From the perspective of obtaining gait physical quantities, both IMUs and gyroscopes provide continuous kinematic data during movement and offer significant advantages for capturing key gait physical quantities. Both sensors can provide temporal parameters, such as stance and swing time. However, gyroscopes specifically offer rotation data across three axes, which contribute to measuring changes in joint angles. IMUs combined with gyroscopes can also provide additional gait quantities, such as stride length and walking speed. These parameters enable a more detailed and comprehensive analysis of gait mechanics, contributing to a deeper understanding of movement patterns.

The main challenges in using gyroscopes and IMUs for gait event detection include processing raw sensor data and effectively removing noise [94,102]. Both accelerometers and gyroscopes are susceptible to various sources of noise, which can impact the accuracy of the gait event detection and the calculation of gait physical quantities. Proper data processing techniques, like signal filtering, are essential to ensure the accuracy of detecting gait events. In addition, other factors, such as temperature variations and magnetic interference, may add additional complexities when it comes to the accurate acquisition of data.

Most studies mention the type of filter used, with the Butterworth low-pass filter being the most common choice for noise reduction and data smoothing. However, the impact of filter settings, such as filter order and cut-off frequency on gait events detection, is rarely discussed [102]. A higher filter order can cause overshooting, while a low cut-off frequency smooths the signal but may result in the loss of critical details. Over-filtering can distort the signal and lead to temporal drift of peaks and troughs, particularly when using peak heuristic algorithms to detect gait events. Currently, few studies have reported or examined the influence of filter settings on gait events detection.

Sensor placement and sensor synchronization are critical considerations for gait analysis based on multiple IMUs [103,104]. Sensors should be positioned as close as possible to the anatomical region of interest, as signals obtained from different locations on the lower limbs typically exhibit distinctive waveform characteristics and temporal variations, resulting in differences in gait spatiotemporal parameters. Applying predefined rules for gait event detection without considering sensor locations may result in decreased accuracy. Additionally, inappropriate sensor orientation can introduce biases; even minor deviations in sensor placement or alignment may distort signal features or waveforms, potentially obscuring critical gait event signatures. Moreover, sensor displacement and vibration during movement, such as soft tissue oscillations or sensor slippage relative to the skin can further compromise measurement accuracy [93]. Sensor synchronization plays an essential role by providing a unified temporal reference across multiple sensor systems. Temporal misalignment among sensors can significantly reduce the precision of gait event detection and adversely affect the accuracy of derived spatiotemporal parameters.

### 4.2. Comparison Between Gait Events Detection Method

In gait event detection, two approaches have been reported: rule-based methods and machine learning methods. Rule-based methods typically rely on predefined rules like thresholds or peak point detection to identify key events based on time domain data. The main advantage of rule-based methods lies in their simplicity and interpretability. Researchers can clearly define criteria for detecting gait events based on target population or specific walking environments, making the detection process easier to understand and verify. These methods have proven effective in the detection of key gait events, particularly in controlled environments with regular, cyclic gait patterns. In the application level, due to the constraints of clinic space, patients may only have a few meters or steps available for gait analysis. The data collected by the sensors in these short sessions is significantly less compared to prolonged monitoring. In such cases, a rule-based approach to gait event detection offers fast and accurate processing of short-term data, introduces less variability, and improves detection accuracy.

However, rule-based methods face limitations when dealing with irregular or pathological gaits, where the consistency of gait cycles is less predictable [43]. They also struggle with more complex gait scenarios, such as curved walking, where simple thresholds may not capture the variability in gait mechanics. Additionally, the effectiveness of rule-based methods depends on the fixed location and orientation of the sensors. Any changes to sensor placement or orientation alter the sensor waveform during walking, making it difficult to apply established rules accurately, which affects the detection of gait events. Although some established rules have been widely cited in many studies [105,106,107] as the basis for detecting gait events, discrepancies still exist in the reviewed articles where sensor positions were supposed to be the same, but the locations of gait events differed on the signal waveform. When applying these rules, researchers must not only consider the position and orientation of the sensors on the lower limbs but also re-examine the use of additional gait event detection devices or methods to ensure the accuracy of gait event positioning in the signal waveform.

Machine learning approaches offer a more flexible, data-driven alternative to rule-based methods. These models learn from large datasets to identify patterns and correlations that are not easily defined by simple rules. The key advantage emphasized in articles implementing machine learning is that machine learning models excel at handling complex and noisy data, for example, irregular gait patterns or gait patterns at different speeds [43], or for people with conditions or pathologies that affect their gait [56,57,90,91]; therefore, they have potential for real-world applications.

In terms of limitations, machine learning requires a large number of well-labelled datasets for training. For example, datasets collected from healthy populations under specific conditions may not be applicable when analyzing pathological gaits, requiring additional data to clearly differentiate the target group from normal populations. Furthermore, these models are often viewed as “black boxes”, making their internal decision-making processes difficult to interpret and explicitly link them with physical gait events.

When comparing the two methods, rule-based and machine learning methods both have strengths and weaknesses in detecting gait events and gait phases. Overall, rule-based methods excel in regular gait and controlled environments due to their high temporal accuracy, while machine learning methods show greater adaptability, especially when dealing with irregular gait and pathological gait. Rule-based methods have been shown to demonstrate efficacy in the determination of gait events with respect to temporal errors, typically within the millisecond range. For instance, one study [63] reported median absolute errors of 7 ms and 8 ms for the detection of HS and TO events, respectively, whereas another study [45] demonstrated mean errors of −1.6 ± 6.3 ms and 0.4 ± 9.0 ms for HS and TO, respectively. Most rule-based methods have been employed in controlled environments, such as the detection of the gait of healthy individuals walking in a straight line. While some rule-based approaches can determine gait events in pathological gait [63], the performance may decline when irregularities or other pathological features are present in multiple gait conditions. This is primarily due to their reliance on predefined thresholds or waveform features, rules that are difficult to adapt to complex, changing signals. Machine learning methods have demonstrated greater adaptability, especially when dealing with irregular or pathological gaits. For example, research [56] has shown that SVM models demonstrate high accuracy (F1 score ≥ 0.95) in distinguishing gait events between healthy individuals and Parkinson’s patients. Conversely, the HMM model has also exhibited the capacity to accurately differentiate between gait phases in both healthy and patient populations [90].

Are machine learning methods superior to rule-based methods in determining gait events or phases? Study [90] compared the two methods for distinguishing gait phases between healthy and patient populations and showed that the overall accuracy using the HMM model was higher than the overall accuracy using rule-based methods for both healthy and pathological populations. However, in study [56], the authors compared the F1 scores derived from the improved SVM model for healthy and Parkinson’s populations with their previously developed threshold-based method. The results showed that the F1 scores of the improved SVM model were slightly lower than those of the threshold-based method. Therefore, based on the findings of the current literature, the strengths and weaknesses of different methods may vary depending on the specific application scenario.

In addition, in terms of computational complexity and data requirements, rule-based methods are particularly suitable for short-time gait detection scenarios with less data, due to their computational simplicity and real-time advantages. In contrast, machine learning methods are more dependent on large-scale, high-quality labelled datasets and require a large amount of data widely covering different gait patterns for labelling and training. Therefore, in practical applications, the selection of these two methods should be weighed comprehensively according to the specific detection goals, gait characteristics, and data conditions, to achieve the best detection results.

### 4.3. Clinical Applications and Future Directions

The use of IMUs and gyroscopes for gait analysis shows significant potential for clinical applications. Previous studies have demonstrated the efficacy of these devices in acquiring physical gait data from patients with neurological and orthopedic conditions [108]. These devices have been shown to accurately detect gait events and measure relevant gait quantities, enabling the characterization of pathological gait types through spatiotemporal data analysis.

Despite this, the review highlights that current studies are generally limited in scope, use only a small number of individuals and fixed laboratory testing environments, with only a few studies using the devices in practical applications and data collection in clinical settings. From a data perspective, the focus of smart sensors has primarily been on anteroposterior direction gait quantities, including time, speed, and step or stride length. Less attention has been given to longitudinal and mediolateral quantities, such as step width and step height.

From an application standpoint, the gyroscope and IMU sensors are small, relatively cheap compared to other measurement systems, and well suited for clinical use. They provide valuable information on pathological progression and the effectiveness of rehabilitation strategies by accurately detecting gait events and relative gait quantities. They are ideal for embedding into foot wearable devices, such as insoles and orthotics, for both short-term clinical examination [34] and long-term monitoring and rehabilitation [109,110]. At the same time, very little information is available to propose standard methods for using gyroscope and IMU sensors in clinical scenarios. Specifically, there is no agreement about the recommended type or number of sensors, sensor placements, or the orientation of the sensor axes. While multiple IMUs or IMUs in combination with other sensors as a tool for gait analysis offer potential advantages, further considerations are required. These include the necessity for sensor synchronization functions and appropriate sensor placement, especially for wearable devices applied in clinic environments. In addition, other influence factors, such as magnetic interference, may add additional complexity and uncertainty, making it difficult to propose best-practice recommendations at this stage. The establishment of best-practice standards is clearly an important goal. These standards should include guidelines for wearable device and system manufacturers for acquiring spatiotemporal parameters and clinical guidelines, as the application of sensors will allow researchers to build comparative databases, and help health providers and doctors apply quantitative measures to assess and diagnose patient data.

The diversification of gait events acquisition is likely to be a future trend for researchers. From a data analysis perspective, the choice between rule-based methods and machine learning approaches often depends on the specific context of the gait analysis. Rule-based methods offer simplicity and transparency, making them suitable for controlled environments with regular gait patterns. In contrast, machine learning approaches provide greater flexibility and adaptability, especially in handling complex or irregular gaits, and are more effective in real-world, uncontrolled environments.

The future of gait event detection may rely in combining these two approaches. Hybrid methods that integrate the interpretability of rule-based systems with the robustness and adaptability of machine learning models could offer optimal solutions for robust gait event detecting methods. Such approaches could provide accurate and reliable gait event detection while maintaining the clarity needed for clinical and research applications.

In terms of sensor diversification, IMUs and gyroscopes combined with other types of instrumentation, such as pressure sensor, EMG, and video analysis, could enhance data richness and improve the overall accuracy of gait assessment in a clinical setting [13]. Integrating these different sensor modalities could also provide a more comprehensive understanding of gait mechanics, leading to better diagnosis and tailored interventions for people with mobility impairments.

## 5. Limitations

This review has some limitations. The search terms employed in this review, i.e., ‘gait events’ and ‘gait phases’, may have unintentionally excluded articles that employed more specific terminology. Expanding the search to encompass additional databases and a broader range of keywords may have enhanced the robustness of this review.

This review focused on the methods for acquiring gait events, gait spatiotemporal parameters using gyroscopes, and IMUs rather than signal processing techniques. Although we briefly discuss the acquisition of gait spatiotemporal parameters, detailed explanation of the technological approach to IMU sensors in terms of sensor fusion and sensor synchronization is still lacking. A previous review [26] reported the technical approach for acquiring gait spatiotemporal parameters using IMUs, and specifically addressed sensor fusion in studies. In addition, studies have provided synchronization solutions between IMUs [111] and IMUs with video [112]. Notably, a review [6] pointed out that proper sensor synchronization for various systems is crucial for continuous real-time monitoring when introducing multiple wearable sensors into the clinic.

It is recommended that future research integrate various aspects, such as data processing pipelines, sensor placement, gait event detection methods, data reliability, and cost benefit analysis, into an efficient data processing workflow to support robust and reproducible detection in a clinical setting.

## 6. Conclusions

This review summarizes current methodologies utilizing wearable sensors—specifically gyroscopes (angular velocity signals) and IMUs (combined angular velocity and acceleration signals)—for the detection of gait events, identification of gait phases, and calculation of spatiotemporal gait parameters. By clearly identifying the contributions for both sensors and analyzing the literature, it is known that gyroscopes are currently employed as the primary tool for detecting gait events, whereas IMUs, integrating both gyroscope and accelerometer data, provide additional robustness and reliability in identifying key gait events. In addition, a variety of analytical approaches and mathematical models exist for gait events and phase detection. Rule-based methods are simple, intuitive, and particularly suited for controlled environments, whereas machine learning methods offer greater flexibility and potential advantages when handling irregular or pathological gait patterns. Furthermore, the data from IMUs contribute not only to gait event detection but also facilitate the computation of spatiotemporal parameters. A variety of sensor fusion techniques combined with biomechanical principles have been proposed and validated within the literature for extracting gait parameters. However, due to varying research objectives and focus, no universally accepted standards currently exist regarding the optimal number of sensors, sensor placement, or filter settings, particularly for practical clinical applications, highlighting the need for further studies to develop standardized guidelines for clinical implementation.

Future research should prioritize the standardization of sensor placement, the developments of robust sensor fusion algorithms, and configuration specifically for clinical application. The exploration of hybrid methodologies integrating the complementary strengths of angular velocity and acceleration signals, and the expansion of clinical applications can further improve the accuracy, reliability, and practical utility of gait assessments. Furthermore, gyroscope and IMU sensors, easily integrated into footwear or ankle-worn devices, present significant opportunities for clinicians and healthcare providers for robust, cost effective, and user-friendly clinical use. Such advancements could address diverse patient needs, enable real-time diagnosis and continuous monitoring, and significantly impact health management, especially within remote or under serviced communities.

## Figures and Tables

**Table 2:** Validation equipment or method and results.

Ref.	Validation Equipment or Method	Results for Gait Events, Gait Phases Detection
[59]	F1-score, temporal error, precision and recall	TCN models demonstrated lower temporal error (20 ms) for both HS and TO events.
[60]	Motion capture system (Vicon system), pressure mat system (GaitRite)	All events (HS, HO) were identified with less than a 2% difference from those obtained with the photogrammetry system
[61]	Force plates	The smallest error in gait event detection was found at IC, and the largest error rate was observed at OTO with an error rate of −2.8 ± 1.5% in the patient group. Overall, the error rates were calculated to be within 3% for all cases.
[62]	Footscan2 plate system (RSscan International Company, Belgium)	The study did not directly state accuracy metrics for gait events, but used the accuracy of spatiotemporal parameters (stride length, step length, stride velocity, and cadence). The mean absolute errors (MAEs) of the stride length, step length, stride velocity, and cadence are 1.77 ± 1.22 cm, 2.40 ± 1.83 cm, 0.02 ± 0.01 m/s, and 1.16 ± 1.37 steps/min, respectively.
[63]	GAITRite^®^ walkway, F1-score, precision and recall	For the results of the health test cohort. The median absolute error is 8 ms for TO and 7 ms for HS. The F1-score, precision and recall are all 100%. Multiple sclerosis and equino varus foot cohorts, with F1-scores of 99.4% and 96.3% respectively, and median absolute errors of 18 ms and 26 ms.
[64]	Accuracy, F1-scores, precision and recall	98.64% accuracy in stance and swing identification
[36]	Force sensitive resistor (FSR), Positive Predictive Value	Average detection accuracy of HS event is 96.64%, with an average detection latency of approximately 20 ms when used DNN model
[55]	Spatial root mean squared error (sRMSE), spatial mean absolute error (sMAE), temporal mean absolute error (tMAE)	Model had a spatial root mean square error of 5.00 ± 1.65%, and a temporal mean absolute error of 2.78 ± 0.97% evaluated at the HS.
[45]	Force plates, motion capture system	The mean errors for each gait event were IC: −1.6 ± 6.3 ms, HO: −0.9 ± 29.3 ms, TO: 0.4 ± 9.0 ms, feet adjacent: −0.5 ± 7.4 ms, and tibia vertical: −0.9 ± 6.4 ms. The range of intra-class correlation coefficient for all gait events had range from 0.896 to 0.998.
[65]	Accuracy, F1-scores, confusion matrices	The study reports 7 gait phases, including LR, MSt, TSt, PSw, IS, MSw, and TSw, and compared with the results of LSTM. Average online accuracy of 98.69% and 97.94% for seen and unseen subjects, respectively.
[66]	Force sensitive resistor (FSR)	The Support Vector Machine (SVM) model using a cubic kernel achieved an accuracy rate of 92.4% when differentiating between gait events using the computed statistical features
[67]	Pressure mat system (GaitRite)	The enhanced gait segmentation algorithm demonstrated greater accuracy than the Salarian gait segmentation algorithm when detecting gait events within one second, for both FO (96% vs. 90%) and FC (94% vs. 91%).
[68]	Force sensitive resistor (FSR)	The overall accuracy of gait phases recognition is 86.43%. The accuracy of different gait phases, such as swing phase: 91.39%, FF: 85.45%, HS: 85.08%, and HO: 83.82%.
[69]	Accuracy, MSE, F1-score, precision and recall	99.96 ± 0.05% for gait event detection. Error rates were around 0.1 to 0.3% on all events
[50]	Force plates	97% accuracy in gait phase estimation for both overground and treadmill walking
[47]	Electromyography(EMG); treadmill force plate (AMTI, USA)	The mean prediction errors for IC and TO events were −4.6 and 2.9 ms. The prediction time range for IC events in the experiments is 50–150 ms, while for TO events, it is 40–180 ms
[70]	Force sensitive resistor (FSR)	The gait event detection success rate for IC was 100% and for TO was 99.72%. The predicted IC and TO events had a mean lead of 8.95 ms and 4.42 ms relative to FSR IC event timing
[71]	Foot switch, motion capture system	N/A
[40]	Force sensitive resistor (FSR), precision and recall, accuracy, sensitivity	N/A
[51]	Force sensitive resistor (FSR), F1-score, precision and recall	High-performance scores (F1-score of gait events ≥0.92). The gait events prediction for HS and TO were about 77 ± 10 ms and 141 ± 10 ms in advance, respectively.
[72]	Force platform, accuracy and macro-F1-score	The accuracy of gait phase recognition reached 97.21%
[44]	Motion capture system (Vicon system)	N/A
[42]	Virtual reality, F1-score, precision and recall	Precision of 83.7 ± 7.7% in gait phase prediction
[38]	Video collection during experiment	Estimation error of MSt was about 7.34% in average
[73]	Motion capture system, accuracy, F1-score, precision and recall	Gait phase segmentation using the TCN-LSTM approach yielded an accuracy of 98.9% with 98.9% and 98.8% for precision and recall. F1-score of 98.9%
[74]	A set of rules using a foot-mounted IMU	The median delays compared with gold standard are HS: −3 0 ms, FF: 40 ms, HO: −10 ms, TO: 0 ms. Median F1-score ≥ 0.955 for all events in intra-subject evaluation
[58]	Force platform (AMTI OR6-7, Advanced Mechanical Technology, Inc., Watertown, MA, USA)	Gait events classifiers returned an accuracy of 91–93%, while the stance vs. swing classifier reached 95.6%. gait events identification returned an average error between −11 ms and 5 ms (95% CI, HS) and between −13 ms and 50 ms TO.
[75]	GRAIL platform with treadmill and force plates	A mean error of −2 ± 9 ms and 20 ± 40 ms for detecting the IC and FC.
[76]	Pressure sensor	The detection of gait events such as HS, HO, and TO obtained 100% accuracy with the IMU. The delay between the estimation of the gait event, and its actual occurrence is upper bounded at 10 ms
[77]	Statistics (ANOVA, post-hoc Tukey–Kramer test, Spearman’s rank-order correlation), video	The largest error between IMUs and the video-based foot impact detection was 0.03 s
[43]	Accuracy	The accuracy of the model in detecting the three gait phases (TO, MSt, HS) was very high (close to 99.5%).
[78]	Motion capture system (Qualisys, Göteborg, Sweden), F1-score, precision and recall, mean absolute error	IMU-based gait event detection showed high recall (IC: ≥97%, FC: ≥96%), high precision (IC: ≥100%, IC: ≥100%) and high F1-score (IC: ≥99%, FC: ≥98%)
[79]	Motion capture system (Qualisys, Göteborg, Sweden)	N/A
[80]	Statistics (mean and root mean square, ANOVA test)	N/A
[81]	N/A	N/A
[82]	Accuracy, F1-score, precision and recall	The accuracy of the gait phase using the HMM model under the three parameter adjustment methods was 91.59%, 91.16% and 91.88% respectively
[41]	Zebris Rehawalk instrumented treadmills	The error (mean ± standard deviation) for the relative stance duration is 1.04 ± 1.34% and swing duration is −1.01 ± 1.35% for healthy subjects
[83]	Absolute difference	Systematic delays were reported for IC: 0.006 s and FC: −0.029 s in lower back algorithm, whereas 0.01 s delay in IC detection was reported for the shank-based algorithm
[57]	Motion capture system (Vicon system)	The mean errors of HS and TO were −14 ms and 23 ms respectively.
[84]	Force plate (GaitRite), accuracy, F1-score, precision and recall, absolute mean difference	The F1-score for healthy subjects was 1. Both precision and recall results were also 1.
[85]	Accuracy, F1-score, precision and recall	Overall, the DCNN detected swing phase with the highest classification accuracy (99.3%), followed by PSw (96.2%), MSt (95.8%). The lowest recognition accuracy was observed for TSt (92.9%).
[86]	Motion capture system (Vicon system)	N/A
[56]	Force sensitive resistor (FSR), F1-score, precision and recall	High accuracy for the healthy group (F1-score: 0.988) and Parkinson’s disease group (F1-score: 0.974) when using heuristic threshold-based method. Average absolute mean difference across the four events was 71 ± 62 ms for healthy group and 57 ± 61 ms for PD group.
[87]	Video, F1-score, precision and recall, mean values of the time differences, absolute mean differences	High accuracy for the three subjects: healthy (F1-score: 0.99), hemiparetic (0.97) and myelopathic (0.96). Detection of HS in advance with an average MD of −44 ms and detected TO with a small delay of 25 ms (average MD across subjects).
[88]	Motion capture system (Optitrack, NaturalPoint, OR, USA) and force plates (AMTI, MA, USA)	Temporal differences for average IC detection is 4.22 ± 15.48 ms, for average TO detection is −8.31± 21.02 ms
[53]	Foot pressure measuring system (T&T medilogic Medizintechnic GmbH)	N/A
[37]	Force plate (Advanced Mechanical Technology Inc., Watertown, MA, USA)	The absolute mean error of IC was 7 ± 3 ms, and the relative mean error was 1.04 ± 0.48%. The absolute mean error of TO was 19 ± 11 ms, and the relative mean error was 2.86 ± 1.62%.
[89]	Motion capture system (Oqus^®^, Qualisys AB, Gothenburg, Sweden)	N/A
[90]	Force sensitive resistor (FSR), true positive rate (TPR) and true negative rate (TNR),	Overall accuracy using threshold was 63.96% for the healthy group and 65.43% for the pathological group. Overall accuracy using HMM was highest 81.44% for the healthy group and 78.06% for the pathological group.
[91]	Force sensitive resistor (FSR)	Accuracy results are above 96.5% for detection gait phases
[92]	Force sensitive resistor (FSR)	The mean accuracy of classifying HS and TO as normal/abnormal is 94.4%
[93]	Motion capture system (OptiTrack Motive Body 1.10, NaturalPoint, Inc., Corvallis, OR)	Average flexion at gait events for HS (5.4° ± 2.3° vs. 10.7° ± 1.4°, *p* < 0.0001), MSt (22.7° ± 3.2° vs. 21.9° ± 4.0°, *p* = 0.54), TO (14.7° ± 1.5° vs. 11.9° ± 3.3, *p* = 0.06), and MSw (72.4° ± 6.8° vs. 69.2° ± 2.8°, *p* = 0.20)
[46]	Motion capture system (Vicon Bonita)	N/A
[94]	N/A	Difference in detection timing was an average of 50 ms late for HS, while TO was detected an average of 35 ms late for TO1 and 70 ms late for TO2
[95]	Force sensitive resistor (FSR)	HS was the gait event most accurately detected under control (accuracy of 100%) and real-life situations (accuracy > 96.98%).
[96]	Force plate system (AMTI, Watertown, USA)	The mean time errors of HS and TO detection are −10 ms and 19 ms.
[49]	Motion capture system (NaturalPoint, Corvalis, USA)	Errors in timing estimation (±0.08 s) of TO events and 0.01 s of IC event.
[52]	MatScan system (Vertical ground reaction force)	N/A
[97]	MatScan system (Vertical ground reaction force)	Negative zero crossing showed good concurrence for HS event timing with a mean difference of −1.5 samples (30 ms) and 72% of gait events.
[98]	Motion capture system (PhaseSpace IMPULSE system)	N/A
[99]	Pressure-sensing insoles (F-Scan 3000E, Tekscan)	100% detection IC and FC when shank method was used.
[39]	Motion capture system (Vicon system)	N/A
[54]	True positive rate (TPR) and true negative rate (TNR)	The highest values of specificity and sensitivity (>0.98) for the three classifiers examined here were obtained when the angular velocity of the foot was processed.
[48]	Statistic (Kolmogorov-Smirnov, ANOVA test, Kruskal-Wallis, Mann–Whitney U test, post-hoc tests)	N/A
[100]	Pressure mat system (GaitRite CIR Systems Inc., Havertown, PA, USA), high-speed video camera system (Canon EX-FH25 Exilim)	Probability of true-positive event detection for the IMU system is 100%, and the probability of false-positive event detection is 0% when referenced to the video system and to the pressure mat.
[101]	N/A	N/A

Note: Due to the different validation criteria and methods, accuracy is reported in different units, as determined by each study. See Table 1 for the methodological details for each study.

## Supplementary Materials

The following supporting information can be downloaded at: https://www.mdpi.com/article/10.3390/s25113481/s1, Table S1: Glossary of the specialized vocabulary [5,61,100,113,114].
