# Supplementary material for: A Review of Gait Analysis Using Gyroscopes and Inertial Measurement Units
Source: Sensors (Basel). 2025 May 31;25(11):3481. doi: 10.3390/s25113481 (PMC12158269; doi:10.3390/s25113481)
Supplement: Supplementary file 1 [file sensors-25-03481-s001.zip › sensors-3618939-supplementary.pdf]

Table S1: Glossary of the specialized vocabulary [1-5]

The terminology for gait events and gait phases		
Vocabulary	Abbreviations	Definition
Gait cycle	GC	The time interval between two successive occurrences of one of the repetitive events of walking.
Stance phase		The entire period during which the foot is on the ground; begins with IC.
Swing phase		The time the foot is in the air for limb advancement, begins as the foot is lifted from the floor (TO).
Initial contact	IC	The phase includes the instant the foot drops on the floor and the immediate reaction to the onset of body weight transfer.
Loading response	LR	The phase follows the IC of the foot with the floor and continues until the other limb is lifted for swing.
Mid-stance	MSt	The first half of the single limb support interval. It begins as the other foot is lifted and continues until body weight is aligned over the forefoot.
Terminal stance	TSt	This phase completes single limb support. It begins with heel rise and continues until the other foot strikes the ground.
Pre-swing	PS	The final phase of stance, the second (terminal) double stance interval in the GC. It begins with IC of the opposite limb and ends with ipsilateral toe off.
Initial swing	IS	The first phase of swing is approximately one-third of the swing period. It begins as the foot is lifted from the floor and ends when the swinging foot is opposite the stance foot.
Mid-swing	MSw	The middle third of the swing period; begins as the swinging foot is opposite the stance limb. The phase ends when the swinging limb is forward, and the tibia is vertical.
Terminal swing	TSw	The final phase of swing; begins with a vertical tibia and ends when the foot strikes the floor.
Heel strike	HS	The moment of heel contacting the floor.
Foot flat	FF	Forefoot contact terminates the heel-only support period and introduces a plantigrade or foot flat posture.
Heel off	HO	Heel rise changes the mode of foot support to the forefoot. This occurs at the 31% point in the GC and persists until the end of stance.
Toe off	TO	The moment of toe lifting the floor, it is common for the toe to be the last segment to lift from the floor at the end of stance.
Final contact	FC	Same as toe off
Foot contact		Same as heel strike
Foot off	FO	Same as toe off
Opposite initial contact	OIC	Same as initial contact
Opposite final contact	OFO	Same as final contact and toe off
Termination of forward swing	TOFS	Termination of forward swing, same as TSw

Initiation of forward swing	IOFS	Same as toe off

1. Perry, J.; Burnfield, J. M., Fundamental. In Gait Analysis Normal and Pathological Function, 2010; pp 3-6.
2. Whittle, M. W., Normal gait. In Gait analysis, 2007.
3. Jacquelin Perry, J. M. B., Normal gait. 2010.
4. Hundza, S.; Hook, W.; Harris, C.; Mahajan, S.; Leslie, P.; Spani, C.; Spalteholz, L.; Birch, B.; Commandeur, D.; Livingston, N., Accurate and Reliable Gait Cycle Detection in Parkinson's Disease. IEEE Trans. Neural Syst. Rehabil. Eng. 2014, 22, (1), 127-137.
5. Yang, S.; Koo, B.; Lee, S.; Jang, D.; Shin, H.; Choi, H.; Kim, Y., Determination of Gait Events and Temporal Gait Parameters for Persons with a Knee-Ankle-Foot Orthosis. Sensors 2024, 24, (3), 964.
